# A single-cell based precision medicine approach using glioblastoma patient-specific models

**DOI:** 10.1038/s41698-022-00294-4

**Published:** 2022-08-08

**Authors:** James H. Park, Abdullah H. Feroze, Samuel N. Emerson, Anca B. Mihalas, C. Dirk Keene, Patrick J. Cimino, Adrian Lopez Garcia de Lomana, Kavya Kannan, Wei-Ju Wu, Serdar Turkarslan, Nitin S. Baliga, Anoop P. Patel

**Affiliations:** 1 Institute for Systems Biology, Seattle, WA USA; 2 Department of Neurological Surgery, University of Washington, Seattle, WA USA; 3 Human Biology Division, Fred Hutchinson Cancer Research Center, Seattle, WA USA; 4 Department of Pathology, University of Washington, Seattle, WA USA; 5 Center for Systems Biology, University of Iceland, Reykjavik, Iceland; 6 Departments of Microbiology, Biology, and Molecular Engineering Sciences, University of Washington, Seattle, WA USA; 7 Brotman-Baty Institute for Precision Medicine, University of Washington, Seattle, WA USA; 8 Present Address: Department of Neurosurgery, Preston Robert Tisch Brain Tumor Center, Duke University, Durham, NC USA

**Keywords:** Systems biology, Tumour heterogeneity

## Abstract

Glioblastoma (GBM) is a heterogeneous tumor made up of cell states that evolve over time. Here, we modeled tumor evolutionary trajectories during standard-of-care treatment using multi-omic single-cell analysis of a primary tumor sample, corresponding mouse xenografts subjected to standard of care therapy, and recurrent tumor at autopsy. We mined the multi-omic data with single-cell SYstems Genetics Network AnaLysis (scSYGNAL) to identify a network of 52 regulators that mediate treatment-induced shifts in xenograft tumor-cell states that were also reflected in recurrence. By integrating scSYGNAL-derived regulatory network information with transcription factor accessibility deviations derived from single-cell ATAC-seq data, we developed consensus networks that modulate cell state transitions across subpopulations of primary and recurrent tumor cells. Finally, by matching targeted therapies to active regulatory networks underlying tumor evolutionary trajectories, we provide a framework for applying single-cell-based precision medicine approaches to an individual patient in a concurrent, adjuvant, or recurrent setting.

## Introduction

GBM is a highly lethal malignancy of the brain that is refractory to standard-of-care (SOC) therapy, which consists of surgery, radiation (XRT), and chemotherapy with the DNA-alkylating agent temozolomide (TMZ)^1–3^. Despite aggressive treatment, median survival is only 14–17 months. Previous studies have shown that GBM tumors are complex ecosystems of normal cell types and diverse malignant tumor-cell states^4–6^. Such intratumoral heterogeneity complicates tumor treatment informed by genomic, epigenomic, and transcriptomic biomarkers of response detected in bulk because these markers may not be ubiquitously present across the entire tumor-cell population.

The functional consequence of such heterogeneity is the existence of a diverse tumor-cell population composed of distinct cellular phenotypes that can divergently respond to both intrinsic and extrinsic pressure. For instance, SOC-treatment may lead to the selection or induction of drug-resistant states^7^ along evolutionary trajectories that are as yet unknown. Thus, tumor composition just after SOC treatment represents a critical timepoint in the evolution of the disease, but is largely understudied because patient tumors are not typically sampled at this time as part of routine clinical care. Consequently, the ‘residual’ disease and corresponding tumor-cell states that ultimately lead to recurrence are poorly understood. To elucidate the evolutionary trajectories of a given patient’s GBM tumor, tractable model systems that accurately reproduce the heterogeneous tumor-cell states at baseline and those that emerge due to SOC-treatment are necessary.

Precision medicine approaches have traditionally focused on mutation-based profiling but have expanded more recently to include bulk-level transcriptional profiling. However, bulk-level analysis obscures underlying intratumoral heterogeneity and the importance of specific cell-state vulnerabilities. Traditional analysis, e.g., differential gene expression, of transcriptional profiles does not necessarily generate mechanistic insights on master regulators that drive cell-state biology. In theory, these master regulators represent important cell-state-specific vulnerabilities that could be exploited for the development of therapeutic strategies.

**Fig. 1:**
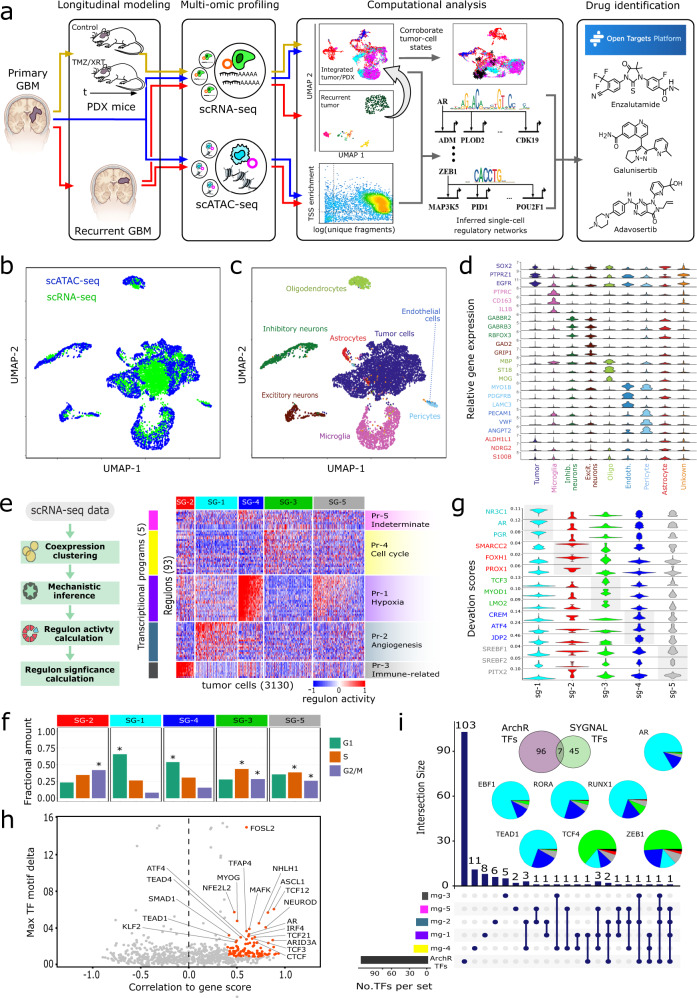
Multi-modal single-cell characterization of UW7 primary tumor biopsy. **a** Schema of overall modeling and analytical framework. UMAP projections of integrated snATAC-seq (4318) and snRNA-seq (3405) profiles. Color annotations indicate (**b**) data type or (**c**) cell type. **d** Violin plots of cell-type marker gene expression (*log*_*2*_*(normalized counts* + *1*)). **e** scSYGNAL/MINER analysis of tumor cells (3130) identified from snRNA-seq profiles from UW7 primary tumor biopsy. Heatmap shows activity levels (z-scores) of regulons (rows) across tumor cells (columns). Regulons are grouped into transcriptional programs (Pr-X) and tumor cells sharing similar regulon/program activity profiles are grouped into transcriptional network states (SG-X). **f** Proportions of SG-X cells in specific cell-cycle phase. Asterisks indicate significant enrichment of cells in a particular cell-cycle phase (FDR *p* value ≪ 0.01). **g** Violin plots of standardized deviation accessibility scores (deviation scores), determined via snATAC-seq, of top three TF binding motifs per network state. **h** Scatter plot of TF motif deviation vs. correlation to inferred gene expression (gene score) via snATAC-seq profiles. Orange points indicate TF regulators whose deviation scores correlate with their corresponding inferred gene expression (gene score) values (correlation ≥0.4, FDR-adjusted *p* value ≤ 0.1) and have a maximum inter-sample group deviation score difference in the upper 50% quantile. Labeled TFs have maximal radial distance from the plot origin. **i** Upset plot outlines number of TFs identified from scSYGNAL and ArchR analysis and number of common TFs shared across all combinations of transcriptional programs and ArchR TF sets. Pie charts indicate the composition of transcriptional network states associated with a positive deviation score for each consensus TFs.

Here, we report a framework developed specifically to model and characterize non-genetic tumor-cell states at various stages of GBM progression including disease onset, recurrence, and the critically understudied periods immediately following SOC-treatment prior to recurrence (Fig. 1a). We applied this framework to an individual patient by performing single-nucleus multi-omic analysis (snRNA-seq, snATAC-seq) of the initial patient biopsy, time-series sample set collected from untreated and SOC-treated patient-derived xenografts (PDX), and the matched XRT-treated recurrent tumor collected at autopsy. We show that the untreated and treated PDX models recapitulated certain phenotypic states observed in the primary and recurrent tumor. Moreover, through the application of systems biology and network inference approaches, we identified multiple candidate mechanistic drivers of treatment-induced transitions in the epigenetic states of tumor cells^6,8,9^. Finally, we applied these network-based insights to identify potential therapeutics that could target specific cell states during various stages of tumor evolution. Together, these results provide a proof-of-concept for how such a framework can inform the rational design of precision therapeutic regimens that account for intratumoral heterogeneity within a given patient.

## Results

### Modeling framework

Our modeling framework used samples derived from a single patient with GBM that was surgically resected, snap frozen, and processed for single nucleus analysis. Biopsy material was also directly xenotransplanted into four NSG mice to generate a cohort of ‘patient avatars’, which were split into either a control (*n* = 2) or treatment (*n* = 2) group (Supplemental Table 1). The treatment group was then harvested 24 h (early) or 72 h (late) post-treatment-completion. These timepoints represent intermediate points in the evolutionary trajectory of the disease that are not routinely sampled as part of clinical care. The patient ultimately reached the terminal stage of disease and was enrolled in a rapid autopsy program that produced snap frozen tissue having a post-mortem interval of 8.75 h. This complete sample set was then integrated into a comprehensive ‘time lapse’ model of tumor-cell states spanning the entire disease process.

### scSYGNAL reveals multiple transcriptional network states in primary tumor cells

We first created a reference landscape from the primary tumor sample by integrating 4318 single-nucleus RNA-seq (snRNA-seq) and 3405 single-nucleus ATAC-seq (snATAC-seq) profiles into a common latent space via uniform manifold approximation and projection (UMAP, Fig. 1b). We then identified 4924 tumor cells (63.7% of the total cell population), characterized by a chromosome 7 gain and chromosome 10 loss, via inference of single-cell CNV state (Supplementary Fig. 1)^5,10,11^ and gene expression markers SOX2, PTPRZ1, and EGFR. The remaining cells were classified into known cell types, based on established gene markers, representing the expected composition of the tumor microenvironment (Fig. 1c, d, Supplemental Table 2).

We then adapted the Systems Genetics Network AnaLysis (SYGNAL) platform^12^ to analyze the snRNA-seq profiles (scSYGNAL) and identify distinct epigenetic programs that were differentially active across tumor-cell subpopulations in the primary tumor. Briefly, using biclustering of snRNA-seq data, we inferred regulons, i.e., sets of genes that share similar expression patterns and are putatively co-regulated by the same set of transcription factors (TFs) or miRNAs across a sub-population of single cells. Our analysis revealed the mechanistic co-regulation of 809 genes across 160 regulons by at least 65 TFs and 141 miRNAs (Supplementary Tables 3, 4). Subsequent analysis via Mining for Node Edge Relationships (MINER) algorithm^13^ revealed a subset of 93 significant regulons that included 52 non-redundant TFs regulating 454 target genes (Methods, Supplementary Tables 3, 4). Of the 52 TFs identified, 33 TFs (63.4%, *p* value = 0.009) have been reported to play biologically meaningful roles in GBM^12,14^, indicating that the single-cell-level network analysis had identified GBM-relevant regulatory mechanisms (Supplementary Table 5). These 93 regulons were further clustered into five metaregulon groups, i.e., transcriptional programs, each characterized by distinct regulon activity profiles across the tumor-cell snRNA-seq profiles (Fig. 1d, e). Comparison of these transcriptional programs with results from unbiased principal component analysis (PCA) revealed that these programs captured major sources of expression variation as regulon activity levels correlated with PC sample scores along multiple PCs (Supplementary Fig. 2). Each program was enriched (FDR *p* value ≤ 0.1) with top-loading genes (largest absolute loading values) along PCs 1 and 2 (Supplementary Table 6). Moreover, expression of top-loading genes within these programs showed a gradient behavior across tumor cells sorted according to their sample scores along PCs 1 and 2 (Supplementary Fig. 2). Finally, distinct biological processes and molecular functions were enriched^15^ within each program (Fig. 1d, Supplementary Table 7), the amalgamation of which we defined as a transcriptional network state.

The primary tumor cells sharing similar transcriptional network states clustered into five sample groups, with each group exhibiting up- or down-regulated activity of specific programs (Fig. 1e). For example, the SG-4 subpopulation expressed increased activity in program 1 (Pr-1), which was enriched for hypoxia-associated genes like *VEGFA*, *PLOD2*, and *PDK1*. Concomitantly, SG-4 exhibited decreased activity of cell-cycle-related regulons (Pr-4) and a corresponding enrichment of non-proliferating cells (Methods, Fig. 1f, Supplementary Table 8)^16^. Similarly, almost every group had some characteristic upregulated program activity (SG-1: angiogenesis, SG-2: immune function and cytokine/interleukin signaling, SG-3: proliferation). SG-5, however, did not exhibit upregulation of any specific program. Rather, these cells expressed varying activity levels of multiple regulons across multiple programs. It is possible that SG-5, which was enriched for proliferating cells (Fig. 1f, Supplementary Table 8), represented tumor cells transitioning between states or tumor cells primed for the expression of various programs.

As these network states represent a novel, regulatory mechanism-based organization of tumor cells, we compared the scSYGNAL-derived network states to two recently established organizing frameworks that characterize GBM tumor-cell heterogeneity and plasticity^4,17^. Categorizing tumor cells along a MES-PN axis^17^ resulted in large proportion (26.5%) of uncategorized tumor cells (Supplementary Fig. 3), suggesting that this framework was not appropriate for characterizing this specific tumor accurately. We then considered a modular framework^4^, which proved to be more appropriate as a much smaller proportion of tumor cells (7.3%) remained uncategorized. We found that the primary tumor cells categorized across all four meta-modules (Supplementary Figs. 3-4). Importantly, each transcriptional network state included cells from all meta-modules, suggesting that scSYGNAL-defined cell states whose regulatory relationships spanned previously defined cell states primarily based on gene expression profiles.

### scSYGNAL and snATAC-seq analysis identifies a network of regulators mediating GBM-relevant gene expression programs in primary tumor cells

Analysis of snATAC-seq data revealed TF motifs enriched in differentially accessible regions of the genome across the tumor-cell population. Using the ArchR package and chromVAR method^18,19^, we identified 141 TFs with differential binding activity across the primary tumor cells (Fig. 1g), 103 of which had motif deviation scores^18^ that correlated with inferred gene expression, i.e., gene scores (Fig. 1h), which provided greater confidence in the putative role of a particular TF in modulating expression behavior of downstream target genes. Comparison of the TFs identified from scSYGNAL/MINER and snATAC-seq analysis revealed a consensus set of seven TFs (AR*, TEAD1, RUNX1, RORA, EBF1, ZEB1, an*d *TCF4)*. Many of these TFs play important roles in tumor biology including *TEAD1* via regulation of YAP/Hippo signaling^20–23^, *TCF4* via regulation of Wnt/β-catenin signaling^24^, *ZEB1* via regulation of epithelial to mesenchymal transition^25,26^, and *AR* via mediation of radiation resistance in GBM^27,28^. Importantly, only a subset of these consensus TFs of interest would have been identified from snRNA-seq analysis alone as only a few were differentially expressed across tumor-cell subpopulations identified via unsupervised clustering (Supplementary Table 9). While the importance of each of these putative regulators in GBM requires further validation, this analysis highlights the value of multi-omic network based approaches, as it was able to identify additional TFs of interest using mechanistic principles of regulation.

The fact that only seven out of 53 TFs were identified by both analyses likely reflects the fact that RNA-seq and ATAC-seq represent different readouts of epigenetic information. snATAC-seq measures regulatory ‘potential’ based on accessibility of chromatin and is not a direct measure of TF binding or downstream activity. Conversely, RNA-seq data is an accurate measure of downstream effects of TF activity. However, individual genes and gene sets can be regulated by multiple TFs, which convolutes interpretation of RNA-seq counts and corresponding TF activity. Consequently, no single modality is capable of accurately and completely predicting the putative TFs driving transcriptional program activity, but the overlap between the two orthogonal modalities provides a consensus set of high confidence regulatory drivers.

### Differential expression and network analysis demonstrate that the PDX tumor avatars replicate primary tumor phenotypic cell states and simulate SOC treatment-induced cell states

We next sought to validate the use of PDXs as a model of heterogeneous cell states in GBM. To do this, we compared primary and PDX tumor cells across multiple levels ranging from gene expression to functional phenotype using various analyses including unsupervised shared nearest neighbor (SNN) clustering^29^, differential expression analysis, statistical enrichment testing, and direct cell-to-cell comparison.

**Fig. 2:**
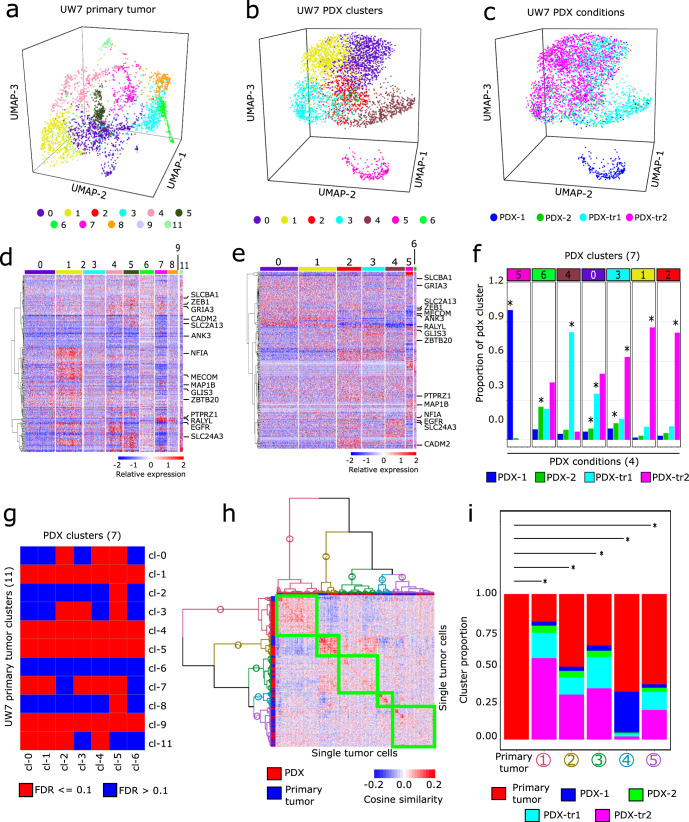
Differential expression and enrichment analysis of primary and PDX tumor cells. **a** 3D UMAP plot of snRNA-seq profiles of UW7 primary tumor cells. Colors indicate SNN-clusters. **b** 3D UMAP plot of snRNA-seq profiles of PDX tumor cells. Colors indicate SNN-clusters. **c** 3D UMAP of PDX tumor cells annotated by their respective treatment condition. **d** Heatmap of normalized gene expression (Methods) of DEGs from the primary tumor cells. The 251 DEGs shown are those genes that have the largest differential expression magnitude in both primary tumor and PDX datasets. **e** Heatmap of normalized expression in PDX samples of the same DEGs depicted in **b**. **f** Proportions of each PDX condition within each SNN-cluster. Asterisks indicate those conditions that are significantly enriched (FDR-adjusted *p* value ≪  0.1) within each SNN-cluster. **g** Heatmap of FDR *p* values indicating significant enrichment of PDX cluster-specific DEGs in primary tumor cluster-specific DEGs. **h** Heatmap of cosine similarity values from pairwise comparison of all primary and PDX tumor cells, based on expression of genes comprising all transcriptional programs. Dendrograms indicate five distinct clusters of tumor cells based on hierarchical clustering. **i** Stacked bar plots show proportion of tumor cell conditions in each cluster. Asterisks indicate hierarchical clusters that have distributions of cosine similarity values, derived from pairwise comparison of all cells within each cluster, that were significantly higher than the distribution from pairwise comparison of only primary tumor cells (FDR *p* value ≪ 0.01).

Dimensionality reduction via PCA and subsequent SNN-clustering revealed that primary tumor cells organized into 11 SNN-clusters associated with 3,531 differentially expressed genes (DEGs) (Figs. 2a, d, Supplementary Table 10) that were enriched for multiple GBM-relevant processes (Supplementary Fig. 5. Supplementary Table 11). Concomitantly, PDX tumor cells organized into seven SNN-clusters associated with 1,731 DEGs (Fig. 2b, e, Supplementary Fig. 6, Supplementary Table 12) that were enriched for similar processes observed in the primary tumor (Supplementary Fig. 5, Supplementary Table 13). Comparison of PDX SNN-cluster membership to experimental treatment conditions revealed that in some cases PDX SNN-clusters were primarily composed of cells from a single treatment condition, whereas in other cases cells from a single condition spanned several PDX SNN-clusters (Fig. 2f). Enrichment analysis confirmed that nearly all primary tumor SNN-clusters (except cluster 6), were enriched with DEGs associated with PDX SNN-clusters (Fig. 2g). In addition, the enrichment of functional gene sets^30–32^ shared across primary and PDX tumor cell clusters suggests phenotypic similarity (Supplementary Information, Supplementary Fig. 7, Supplementary Table 14). Together these results support the ability of the PDX models to simulate, to an extent, primary tumor-cell states in the context of this particular patient.

We then directly assessed similarities and differences between the primary and PDX tumor samples on a cell-to-cell basis with respect to transcriptional program gene expression. Hierarchical clustering of tumor cells based on their cosine similarity profiles produced five clusters, each composed of primary and PDX tumor cells (Fig. 2h, i). Hypothesis testing revealed that all five clusters had a significantly higher distribution of pairwise cosine similarity values between PDX tumor and primary tumor cells than that obtained from pairwise comparison of primary tumor cells only (*p* values ≪  0.05; Fig. 2i). Moreover, projection of PDX tumor cells into PC score and UMAP embedding space, defined by primary tumor cells, showed that the PDX cells overlapped with the primary tumor cells (Supplementary Fig. 5), corroborating similarities between PDX and primary tumor cells within the context of this particular patient.

Finally, examination of underlying functional regulatory mechanisms including regulon gene membership and transcriptional network states showed additional similarities between primary and PDX tumor cells (Supplementary Fig. 8). In particular, comparison of transcriptional network states of primary tumor cells (batch-integrated dataset, Supplementary Fig. 9) to those inferred from untreated PDX samples (non-integrated dataset) via cosine similarity showed that untreated PDX tumor cells exhibited transcriptional network states similar to 44.8% of all network states observed across primary tumor cells (Supplementary Fig. 10), further corroborating the ability of the PDX tumor cells to simulate cell states observed in the patient’s primary tumor.

### Longitudinal categorization of tumor cells reveals distinct dynamic behavior of network modules

**Fig. 3:**
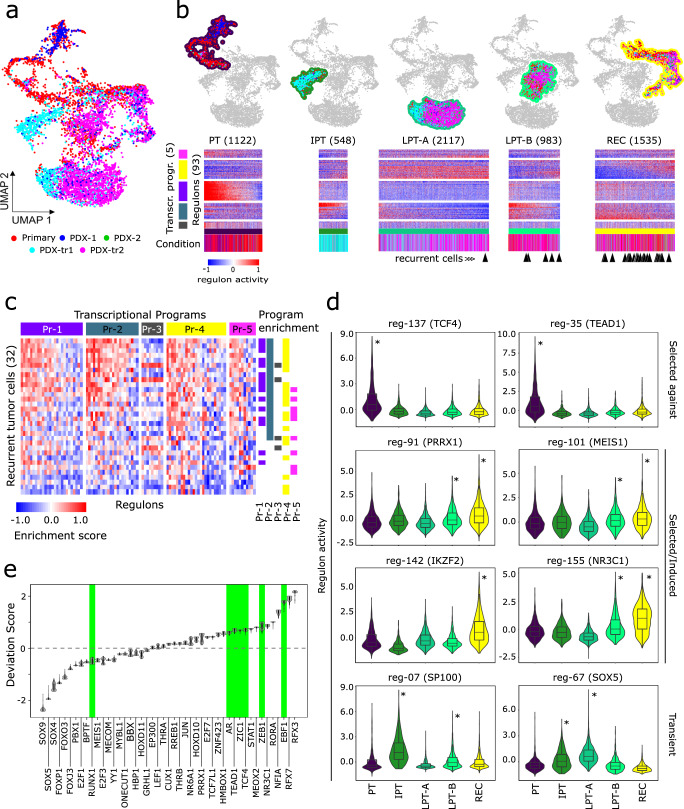
Modeling tumor progression and tumor response to standard of care. **a** UMAP plot of integrated snRNA-seq profiles of UW7 primary tumor (3130) and PDX tumor cells (4388). Colors indicate treatment conditions (Primary: tumor biopsy, PDX-1/2: untreated samples collected at 24 h and 72 h post-treatment of corresponding XRT/TMZ-treated conditions, respectively, PDX-tr1/tr2: samples collected 24 h and 72 h, post-TMZ/XRT treatment, respectively). **b** Four main longitudinal stages (PT pre-treatment, IPT immediate post-treatment, LPT late post-treatment- (**a**, **b**), REC recurrent). Heatmaps show corresponding regulon activity z-scores across tumor-cell subpopulations. Color bars indicate experimental condition of cells as described in **a**. Black arrowheads underneath color bars indicate nearest primary/PDX tumor cell neighbor to recurrent tumor cells, which were co-embedded in the UMAP space (Supplementary Fig. 13). **c** Gene set enrichment analysis of regulon gene sets in recurrent tumor cell snRNA-seq profiles. Top color bar indicates transcriptional programs. Right adjacent color bars indicate samples statistically enriched with regulons for a particular transcriptional program (FDR-adjusted *p* values ≤ 0.1). **d** Violin and embedded boxplots of regulon activity across longitudinal stages. Selected regulons exhibit distinct activity patterns characteristic of “selected-against”, “selected/induced”, or “transient” behavior. Centerline and bounds of the box for each boxplot represent the 50th, and 25th/75th percentile of the cosine similarity values, respectively. Whiskers capture ±1.5 * interquartile range. Asterisks indicate stages where regulon activity is significantly higher relative to the rest of the primary/PDX tumor-cell population (FDR-adjusted *p* value < 0.01). **e** Violin plots of deviation scores across recurrent tumor snATAC-seq profiles. Green background highlights seven consensus TFs (Fig. 1i).

To determine the mechanistic underpinnings of SOC-treatment-induced changes in tumor-cell states over time, we collected samples of untreated/treated PDX avatars at timepoints immediately following treatment, which are not clinically available for this stage of disease progression. Analysis and visualization by UMAP showed that the batch-integrated single-cell transcriptomes of the primary and PDX tumor cells, which showed a high degree of similarity to the non-integrated versions of the datasets at both the regulon and transcriptional-program levels (Supplementary Fig. 8), intermingled across several broad cell groups (Fig. 3a). We identified four broad groups of tumor cells (Fig. 3b), informed by SNN clustering and enrichment analysis of PDX treatment timepoints within the SNN-clusters (Supplementary Fig. 9). Based on the enrichment of timepoints, we ordered the groups into longitudinal stages (Supplementary Fig. 11). The pre-treatment (PT) stage was enriched for cells from the primary tumor and untreated PDX conditions. The “immediate post-treatment” stage (IPT) was enriched for PDX samples collected from the 24-h post-treatment timepoint. The “late post-treatment” (LPT) stage was divided into two subpopulations: LPT-A, which was enriched for samples from both 24-h and 72-h post-treatment timepoints, and LPT-B, which was enriched for PDX samples from just the 72-h post-treatment timepoint. Finally, the “recurrent” (REC) stage included 72-h post-treatment PDX samples, onto which a majority of the recurrent tumor cells (Supplementary Fig. 12) projected in the co-embedded UMAP space (Fig. 3a, Supplementary Fig. 13). This overlap was corroborated by significant enrichment of regulons and transcriptional programs that were active in 72 h post-treatment PDX samples (PDX-tr2) within the recurrent tumor cells (Fig. 3c) as well as by phenotypic similarity between the recurrent tumor cells and those belonging to the REC stage (Supplementary Fig. 14).

It was important to delineate the longitudinal stages in this manner because unsupervised SNN clustering identified clusters mixed with cells from different timepoints. This is consistent with the fact that the evolutionary trajectories as a consequence of treatment and disease progression represent a mixture of “selected” cell states that become enriched or depleted over time and “induced” cell states that are not abundant or present at disease onset, but likely emerge in response to intrinsic and extrinsic factors.

### scSYGNAL/Open Targets platform analysis identifies drugs targeting selected/induced and recurrent cell states

We next investigated whether the scSYGNAL-derived network understanding could enable a therapeutic strategy using drugs that putatively target active transcriptional programs across tumor-cell states along evolutionary trajectories through which tumor cells escape treatment (Supplementary Table 15). Using the Open Targets platform^33^, a curated database of drug-target pairings, we identified drugs targeting TFs or downstream genes in regulons and transcriptional programs having distinct longitudinal activity patterns across the PT, IPT, LPT, and REC stages (Supplementary Table 16). The first distinct pattern identified, labeled as ‘selected against’, was characterized by regulons expressing high activity early in the disease and low activity post-treatment, such as those regulated by *TEAD1* and *TCF4* (Fig. 3d). This pattern suggests that SOC-treatment effectively decreased network activity of these regulons. The second pattern observed described regulons having increased activity in the REC stage (“selected/ induced”), and informed us of potential targets for salvage therapy at the time of recurrence. *PRRX1*, which has been associated with TGF-ß signaling^34^, could potentially be targeted with galunisertib (Fig. 3d). *IKZF2* and its corresponding regulon genes also showed increased activity in the REC stage (FDR *p* value = 0.00, Fig. 3d and Supplementary Fig. 11). Accordingly, *IKZF2* can be targeted indirectly by CDK4/6 inhibitors that target CDK4 activity, which was downstream of *IKZF2* (Supplementary Table 16).

A unique strength of this modeling framework is that it enables the identification of regulons and cell states that are transiently induced during or immediately following treatment. As these cell states likely represent ‘residual’ disease that lead to tumor recurrence, they are of clinical importance. Unfortunately, because activity transiently arises at a time when clinical sampling rarely occurs, these states are missed from typical clinical specimen analysis. However, in our framework transient states were represented clearly by the IPT and LPT stages. *SP100*, reported to play a prominent role in GBM^35,36^ and a TF driver for regulon-7, displayed such transient behavior (Fig. 3d). Regulon-7 showed increased activity in PDX cells enriched in the IPT stage and was enriched in upregulated DEGs specific to 24 h post-SOC-treatment PDX cells (*p* value = 8.13e-04). Moreover, regulon-7 was not enriched for upregulated DEGs associated with any other timepoints or stages. Interestingly, regulon-7 included *PDGFC* and *MEOX2*, which have been associated with poor prognosis and GBM aggressiveness^37,38^. *SOX5*, a TF for regulon-67 (Fig. 3d), was another regulator with similar transient activity patterns. In this case, Open Targets analysis identified the TGF-β pathway^39–41^ and *TTK*^42^ as downstream druggable targets for regulon-67. Transient states governed by TFs such as *SP100* and *SOX5* are appealing targets for concurrent therapies that could be trialed in conjunction with SOC, or as adjuvant therapies after SOC has been completed but prior to recurrence.

Finally, we were also able to identify a number of TFs of interest, including *RFX3* and *RFX7* (Fig. 3e) from snATAC-seq analysis of the recurrent tumor autopsy specimen. These two TFs play roles in ciliogenesis in the central nervous system, an important pathway in regulating GBM growth and resistance to therapy^43^. While *RFX3* was differentially expressed across the tumor-cell populations, *RFX7* was not and would have been missed using snRNA-seq analysis alone (Supplementary Table 17), further highlighting the value of multi-omic data analysis in identifying potential TF targets.

## Discussion

In this study, we developed a modeling and analytical framework to investigate, at single-cell resolution, GBM progression in an individual patient from early disease onset (primary tumor), post-SOC-treatment (PDX mice), and eventual recurrence (recurrent tumor collected at autopsy). We used single-cell, multi-omic profiles, in combination with a priori knowledge of cell types/states and regulatory relationships, to define tumor-cell-state composition and delineate how that composition changed in response to SOC-treatment. These results provide a comprehensive view of the evolutionary trajectory underlying GBM progression within a patient, including the understudied stage immediately following SOC-treatment.

Differential expression, functional enrichment, and direct comparative analysis of clusters identified in the primary and PDX tumor cells revealed similarities among specific primary and untreated PDX tumor cell clusters observed across multiple levels ranging from gene expression, to functional phenotype. While these findings showed that PDX mice models recapitulated multiple phenotypic characteristics of a primary tumor, it is important to interpret these results in the context of the single patient from which these models were derived. Although this framework is singularly focused on an individual patient, these results support the possibility of using PDX models on a broader scale and warrants further study to confirm the consistent accuracy of such models required for general use in clinical care. This is conceptually important as model systems that faithfully represent the heterogeneity seen in patient tumors are currently lacking.

Concomitantly, we applied the network-inference methodology SYGNAL^44^ to infer the underlying regulatory relationships at single-cell resolution (scSYGNAL). This is the first application of the SYGNAL approach in the single-cell context. Through this analysis, we were able to infer regulatory network programs and define tumor-cell subpopulations based on common regulatory mechanisms and similar regulatory network states. Importantly, these network states spanned multiple SNN-clusters and previously established meta-module cell states^4^. In theory, therapeutic strategies targeting regulatory mechanisms have the advantage of potentially targeting multiple cell states simultaneously. As such, our method represents an alternative approach to cell state identification based on gene expression alone. Further experimental validation is required to determine which method is more robust, requiring the use of functional assays to determine the prevalence of tumor-cell subpopulations defined by the different methods. It is also important to note that many of these potential regulators may not be directly druggable and will require techniques like CRISPR-Cas9 or antisense RNA-mediated targeting to validate their regulatory roles. Regardless, insights into inferred regulatory mechanisms and their activity throughout disease/treatment progression enabled us to identify TFs of interest and potential therapeutic strategies having plausible clinical relevance for this particular patient.

### Model limitations and future work

While this framework represents a deep characterization of GBM tumor-cell-state evolution in an individual patient, experimental testing is required to validate the predicted drug-target pairings. The next logical step in this approach is to test predictions of putative therapeutic activity in relevant model systems. Due to limited biopsy sample availability, we were unable to test these predictions in an appropriate, patient-specific model system in this case. Ideally, this would have involved continuous maintenance of a corresponding PDX model (via serial transplantation), an important future direction for this work. Equally important is the systematic characterization of the faithfulness of serially transplanted PDXs to the original patient tumor, which has yet to be performed and requires further study. Nevertheless, predicting therapeutic efficacy using systems biology-based analysis of single-cell data shows promise, but needs to be fully validated and will require larger studies using complex model systems.

**Fig. 4:**
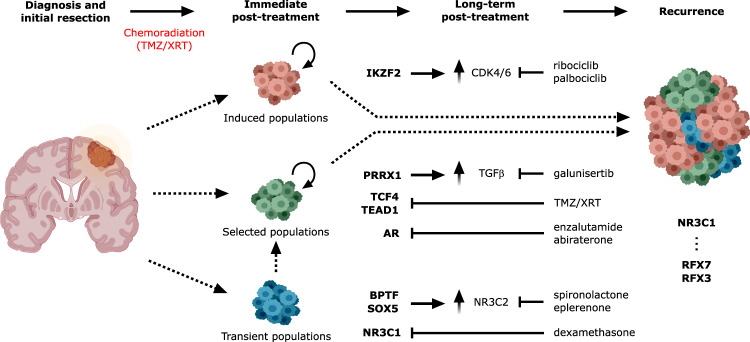
Comprehensive schematic of glioblastoma progression. Distinct populations of “induced”, “selected”, and “transient” tumor-cells states, regulons, and TFs (bold) contribute to intratumoral heterogeneity, which plays a role in treatment resistance. As cell states may be differentially susceptible to treatment and may be selected for or induced by therapeutic intervention, use of a more complete view of cell state trajectories with scSYGNAL and MINER analysis may allow for the prediction of therapies that work in either the concurrent setting against cell states or an adjuvant/neo-adjuvant setting against induced cell states. Figure components were created via BioRender.com.

To our knowledge, this is the first study that infers tumor evolutionary trajectories from single-cell analysis of a comprehensive patient-specific sample set (primary tumor biopsy, treated patient avatars, and a matched recurrent tumor), and subsequently translates these results into potential therapeutic insights. The proof-of-concept work presented herein supports the use of a modeling and analytical system to characterize the spatiotemporal heterogeneity of an individual patient’s tumor throughout disease progression, particularly during stages post-SOC, and inference of potential therapeutic vulnerabilities (Fig. 4). Ultimately, the information gathered from this type of approach could inform clinical treatment in a more targeted, rational manner and enable precision medicine that addresses intratumoral cell heterogeneity and cell-state evolution.

## Methods

### Tumor acquisition

Based upon institutional review board (IRB)-approved protocols (protocol #STUDY00002162), intraoperative tumor specimens from adult patients who voluntarily consented to donation to the institutional tumor bank were collected in cryogenic vials (Corning; Corning, NY) and immediately snap frozen in liquid nitrogen. All patient specimens were anonymized prior to processing. Tumor pathology and diagnosis was confirmed by a neuropathologist as WHO grade IV glioblastoma, IDH-wild type. The specimen was subsequently stored in −80 °C freezers for further experimentation. Autopsy tissue was collected after informed consent with a waiver from the University of Washington IRB with a post-mortem interval of approximately 8.75 h. Tissue was snap frozen in liquid-nitrogen cooled isopentane. Tumor regions were sampled based on gross examination of brain sections and processed as outlined below.

### Tissue processing

Frozen tissue was processed to nuclei using the Frankenstein protocol from Protocols.io. Briefly, snap frozen glioblastoma tissue was thawed on ice and minced sharply into <1 mm portions. 500 μl chilled Nuclei EZ Lysis Buffer (Millipore Sigma, NUC-101 #N3408) was added and tissue was homogenized 10-20 times in a Dounce homogenizer. The homogenate was transferred to a 1.5 ml Eppendorf tube to which 1 mL chilled Nuclei EZ Lysis Buffer was added. The homogenate was mixed gently with a wide bore pipette and incubated for 5 min on ice. The homogenate was then filtered through a 70 μm mesh strainer and centrifuged at 500 g for 5 min at 4 °C. Supernatant was removed and nuclei were resuspended in 1.5 mL Nuclei EZ lysis buffer and incubated for 5 min on ice. Nuclei were centrifuged at 500 g for 5 min at 4 °C. After carefully removing the supernatant, nuclei were washed in wash buffer (1x PBS, 1.0% BSA, 0.2 U/μl RNase inhibitor). Nuclei were then centrifuged and resuspended in 1.4 ml wash buffer for two additional washes. Nuclei were then filtered through a 40 μm mesh strainer. Intact nuclei were counted after counterstaining with Trypan blue in a standard cell counter.

### Animal models

All animal procedures were performed in accordance with protocols approved by the Institutional Animal Care and Use Committee (IACUC) at Fred Hutchinson Cancer Center and the University of Washington. Animals were housed at a maximum of five per cage with 14-h light/10-h dark cycle with food and water ad libitum. Female 4–8 week-old NOD-SCID mice (NOD.Cg-Prkdcscid Il2rgtm1Wjl/SzJ, Jackson Labs; Bar Harbor, ME) were used for all experiments with random assignment into treatment groups where applicable. Mice were monitored at least three times weekly for weight loss and other signs of neurologic or physical distress.

### Patient-derived xenograft modeling

Fresh surgically resected tumor sample was placed in sterile phosphate buffered saline and transported to Fred Hutchinson Cancer Center for further processing. Tumor specimen was dissociated with the use of a papain-based tumor dissociation kit (Miltenyi Biotec, 130-095-942) as per manufacturer’s instructions. Intracranial orthotopic transplantation of single-cell suspension human glioblastoma tumor cells into murine mouse models were performed in standard, IACUC-approved fashion. Briefly, mice were induced with 5% isoflurane and maintained at 2% isoflurane in oxygen thereafter. After appropriate placement on a stereotactic frame (Stoelting Co.), the skull of the mouse was exposed through a small skin incision, and a small 1 mm^2^ burrhole was placed shortly behind and lateral to bregma using a 25-gauge needle. Freshly dissociated cells were suspended in 5 mL of PBS and loaded into a 33-gauge Hamilton needle syringe. The cells were then subsequently injected 2.0 mm lateral and posterior to the bregma and 2 mm deep to the cortical surface. After completion of injection, the syringe was left in situ for another minute before removal in attempt to minimize risk of cell reflux. After scalp closure with suture, the mice were removed from anesthesia and allowed to recover on warming pads and returned to their cages following full recovery. Mice were then checked daily for five consecutive days for signs of distress or neurologic disability. Mice were also monitored using a small animal 1.5 T MRI to track the degree of intracranial tumor, initially four weeks following injection and then again upon signs of neurologic symptoms, including ataxia, head tilt, seizures, or cachexia. Mice were sacrificed as soon as they demonstrated symptoms, and their brains were collected directly following euthanasia.

### Radiation and TMZ treatment

Tumor-bearing mice, as confirmed by small animal MRI, were given 50 mg/kg of temozolomide dissolved in 5% DMSO/saline or vehicle intraperitoneally for five consecutive days. On the same days, tumor-bearing mice were sedated with ketamine and xylazine and irradiated using a X-RAD 320 from Precision X-Ray at 115 cGy/min as has been performed previously^45^.

### 10x Chromium snRNA-seq & snATAC-seq

Single-nucleus RNA sequencing was performed using the 10X Chromium v2 system. Briefly, nuclei were isolated, hashed according to timepoint (see below), and pooled prior to loading on a 10X Chromium chip using single cell RNA-seq 3’ v3 chemistry (SingleCell RNA ReagentKits v3.0 UserGuide RevC) with a targeted cell recovery of 12,000. Gel emulsions were recovered from the microfluidic chip and full-length cDNA libraries were fragmented, end repaired, A-tailed, and ligated to 10X read 2 adapters prior to SPRI cleanup. Final indexing PCR with P5 and P7 Illumina primers was performed as per standard protocols and libraries were quantified using TapeStation D1000, pooled, and sequenced on an Illumina NovaSeq 6000 to a read depth of 50,000 reads per cell using paired end 28:8:0:91 read lengths. snATAC-seq was performed per manufacturer instructions (SingleCell ATAC ReagentKits v1.1 UserGuide RevD). Briefly, nuclei were isolated as already described and transposed in bulk using standardized amounts of Tn5 per 10X protocols. Nuclei were diluted to an appropriate concentration to target a recovery of 5000 and loaded on a 10X Chromium ATAC Controller Chip. Resulting transposed and single-cell barcoded DNA was then denatured and linearly amplified, followed by SPRI cleanup and Illumina P5 and P7 indexing as per standard protocol. Libraries were quantified using TapeStation D1000, pooled and sequenced on an Illumina NovaSeq 6000 to a read depth of ~50,000 reads per using paired end 50:8:16:50 read lengths.

### Cell hashing and demultiplexing

Single nuclei from each PDX condition were labeled with 1 μl condition-specific hashtag oligonucleotide-labeled antibodies (BioLegend, TotalSeq A0541-A0545) according to manufacturer’s protocol prior to pooling and loading on a single lane of the 10X Chromium v2 system. The HTO library was processed separately and spiked in at 10% of the mRNA library prior to sequencing. Demultiplexing of pooled single-cell samples relied on subsequent HTO raw counts generated from snRNA-seq to classify computationally single-cells in their appropriate experimental condition. Demultiplexing was performed using the *HTODemux* function in the Seurat v3.2.2 platform^29^. The resulting single-cell annotation indicated experimental conditions and potential doublet or untagged state of a cell.

### Doublet prediction

For those cells not processed using cell hashing, i.e., UW7 primary and UW7 recurrent autopsy cells, an alternative, computationally-based approach known as DoubletDecon was used to identify likely doublet samples^46^. Briefly, DoubletDecon generates synthetic doublets by merging transcriptional profiles from randomly selected pairs of cells belonging to distinct cell clusters identified in the dataset. These synthetic doublets are used, in conjunction with the previously identified clusters to create a deconvolution cell profile (DCP) for the entire cell population. Pearson correlations are then calculated between each DCP and the centroid of each cluster. Those cells having the highest correlation to clusters comprised of synthetic doublets are labeled as doublets. Prior to final labeling of cells, a rescue step is performed in which certain cells may avoid doublet labeling if the cell contains statistically significant upregulated expression, relative to a synthetic doublet cluster, for a minimum number of genes. Cells meeting that criteria are reincorporated into the non-doublet population. Finally, due to the random nature of synthetic doublet, it is likely that doublet predictions will vary run-to-run. Therefore, we conducted 50 runs to identify a consensus set of predicted doublets, which were subsequently excluded from downstream analysis.

### Quality control and snRNA-seq data pre-processing

We initially processed the 10X Genomics raw data using Cell Ranger Single-Cell Software Suite (release 3.1.0) to perform alignment, filtering, barcode counting, and UMI counting. Reads were aligned to the GRCh38 reference genome using the pre-built annotation package download from the 10X Genomics website. We then aggregated the outputs from different lanes using *cellranger aggr* function with default parameter settings.

Each sample set analyzed via snRNA-seq (UW7 primary-, UW7 PDX-, and UW7 recurrent-tumor samples collected at autopsy) was QC-filtered separately prior to data integration, as in the case of primary and PDX tumor samples, and/or subsequent downstream analysis. Each sample set consisted of the following: 5082 cells with 27,763 mapped genes (UW7 primary tumor), 11,648 cells with 26,231 mapped genes (UW7 PDX samples), and 690 cells with 19,917 mapped genes (UW7 recurrent autopsy tumor). To minimize inclusion of poor-quality genes and single-cell samples, we applied the following QC filters: (1) mitochondrial genes must comprise ≤ 20% of the number of uniquely mapped genes/cell, (2) total counts/cell should be ≥500 and ≤50,000 (UW7 primary tumor cells), ≥500 and ≤24,000 (UW7 PDX samples), or ≥500 and ≤4000 (UW7 recurrent autopsy tumor), and (3) the total number of mapped genes should be ≥500 genes and ≤10,000 (UW7 primary tumor cells), ≥500 genes and ≤7000 (UW7 PDX tumor cells), or ≥500 genes and ≤30,000 (UW7 recurrent autopsy tumor). Following QC-filtering, each sample set consisted of: 4456 cells expressing up to 19,228 genes (primary tumor), 4388 expressing up to 26,231 genes (PDX tumors), and 350 cells expressing up to 12,463 genes (recurrent tumor).

### Data normalization of snRNA-seq data

We applied the *SCTransform* function, provided in the Seurat v3.2.2 platform, to normalize and variance-stabilize UMI counts in the single-cell data. This function develops a regularized negative binomial regression model to characterize the UMI count distribution on a gene-by-gene basis. This model is then used to determine Pearson residuals, i.e., the square root of the variance-normalized difference between the actual gene count and model-predicted counts. These residuals represent the standardized expression values not affected by technical artifacts and were used for downstream analysis. Concomitantly, mitochondrial gene expression influence was regressed out of expression for each gene in each cell, as part of the SC-normalization procedure.

### Cluster identification and analysis of differentially expressed genes (DEGs) of primary-, PDX-, and recurrent-tumor snRNA-seq data

After quality control and filtering, SC-normalized data for all 6867 genes common to the primary- and PDX-data sets and 6541 genes common to the recurrent tumor data were used for dimensionality reduction via principal component analysis (PCA). The first 30 principal components were used as a basis to create a shared nearest neighbor (SNN) graph of the single-cell samples. From this graph, clusters of single cells were identified via Leidan clustering of nodes, i.e., single cells, from the SNN graph.

To identify DEGs in each of the SNN-clusters identified across the primary tumor and PDX single-cell samples, the FindMarkers function in Seurat was used. In particular, the Wilcoxon rank sum test was used with the following cutoff values to identify DEGs: absolute fold change ≥*log2* (1.25), minimum proportion of cells of interest expressing the gene for consideration = 0.1, and an FDR-adjusted *p* value of 0.1. In the case of the recurrent tumors, DEGs were determined based on expression between cells identified as tumor cells (refer to snRNA-seq cell-type and tumor cell annotation section) and non-tumor cells.

### snRNA-seq cell-type and tumor-cell annotation

Established CNS cell-type-specific genes were used to determine gene set module scores for each cell. Gene module scores were determined using the *AddModuleScore* function provided in Seurat. In brief, the module score represents the difference between the mean expression of the gene set of interest and the average expression of a randomly selected set of control genes. To create a control gene set, all genes are grouped into 25 bins according to their respective average expression. Next, for each gene in the gene set of interest, a corresponding set of 100 randomly selected genes is selected from the same expression bin. This results in a control set that is 100-fold larger in size, which is analogous to averaging over 100 randomly selected gene-sets identical in size to the gene set of interest. Positive module scores indicate that the gene set of interest has higher expression than what is expected by random chance and vice versa. Final cell-type assignment was based on which corresponding gene set resulted in the highest positive module score above a threshold value of 0.05. To annotate tumor cells, inferCNV^11^ was used to infer the copy number variation state of each cell (Supplemental Text). Both cell-type and tumor cell state, defined by Chr7 gain and Chr10 loss, were used to determine final cell-type annotation for the primary and recurrent tumor biopsy samples (Supplementary Figs. 1, 12).

Module scores were determined for each meta-module (MES1, MES2, AC, OPC, NPC1, and NPC2) as defined by Neftel et al. (2019). Initially, each cell was assigned a meta-module state based on the maximum score, as described previously. To simplify tumor-cell annotation, cells annotated as either MES1 or MES2 were annotated simply as “MES”. Likewise, cells annotated as either NPC1 or NPC2 were annotated simply as “NPC”.

Within the MES-PN axis of differentiation framework^17^, we defined three gene sets to determine whether a cell was of the MES, PN, or intermediate (INT) subtype. Each category was defined by genes selected according to their PC1 loading values, MES genes were defined as those genes having the highest 100 loading values. PN genes were defined as those having the lowest 100 PC1 loading values. The remaining 514 genes were used to define the intermediate (INT) state. To confirm that the genes used to define the MES and PN states, we compared those loading genes with DEGs associated with each state^17^ and found that the highest loading genes overlapped with the MES DEGs while none overlapped with the PN DEGs and vice versa. We determined module scores and cell annotation using gene sets defined solely on their PC1 loading values as well as gene sets defined by both PC1 loading values and DEGs. We found minimal differences in the proportion of cell types. Because the MES-PN axis^17^ was defined by top-loading genes along PC1, we too defined gene sets based on PC1 loading values. Finally, PC loadings determined from analysis of single-nucleotide RNA-seq (snRNA-seq) were used so that a comparable determination of the MES-PN state could be made from our snRNA-seq single-cell profiles.

### snRNA-seq multivariate analysis

Downstream analysis of snRNA-seq data was performed using Seurat v3.2.2. Following QC filtering, SC-normalization and integration, we performed principal component analysis (PCA) on the integrated gene expression matrix using the first 30 principal components (PCs) for clustering and visualization. Next, we used the transformed gene expression data along the top 30 PCs to identify shared nearest neighbors (SNN). We then identified clusters in an unsupervised manner using Leidan clustering (resolution = 0.8). Visualization of PC scores from the top 30 PCs was performed using UMAP (minimum distance = 0.2, spread value = 1.2).

### Semantic similarity analysis and determination of phenotypic state

To determine functional similarities of regulons and transcriptional programs determined from the batch-integrated and non-integrated primary and PDX tumor cell snRNA-seq datasets, we determined semantic similarity scores as a measure of functional similarity. Briefly, semantic similarity scores between sets of genes were determined via graph-based methods that compare the topology of the GO-term graph structure, where a directed acyclic graph (DAG) includes the term of interest, all related ancestor terms, and the set of edges that connect the GO terms in the DAG. To compare multiple sets of regulon genes, the *mclusterSim* function in the GOSemSim package in R was used. Specifically, the “Wang” method was used to compare quantitatively the DAGs of GO terms associated with each regulon gene set. It is possible that multiple GO terms can be associated with a gene set(s). The result is a matrix of similarity scores. To aggregate these scores, we used the best-match average (BMA) strategy, which calculates the average of the maximum row and column similarity scores from the matrix of similarity scores.

To determine the phenotypic state of a cell, we performed enrichment analysis of MSigDB gene sets^30–32^, which covered a wide range of biological functions, pathways, and oncogenic signatures, within a particular cluster or grouping of tumor cells. Specifically, the hallmark, C2 (curated), C4 (cancer-oriented microarray data), C5 (ontology), C6 (oncogenic signatures), and C7 (immunologic) gene sets were used. Enrichment analysis of DEGs and transcriptional programs were performed using the *compareCluster* function in the clusterProfiler package in R^15^. Statistical enrichment of a gene set was based on an FDR-adjusted *p* value cutoff of 0.1, using the set of 6867 genes shared between the primary tumor and PDX datasets and 6541 genes shared with the recurrent tumor dataset.

Results from the enrichment analysis were combined to produce a vector of 7551 FDR-adjusted *p* values associated with each gene set for each tumor cell cluster (FDR-adjusted *p* value ≤ 0.1). We then converted the adjusted *p* values by taking the *−log*_*10*_*(FDR-adjusted p value)* for subsequent comparison. Gene sets that did not have any overlapping genes with DEGs or those included in transcriptional programs produced an NA result. Because a large majority of gene sets tested did not have any overlapping genes with DEGs or transcriptional program genes, we focused on the top 200 enriched gene sets having the largest row sum of the converted FDR-adjusted *p* value (Supplementary Fig. 7). That is, we focused on the top 200 gene sets enriched across the most tumor cell clusters. The resulting profile of the significantly enriched gene sets defined the phenotypic state of a cell cluster. We performed subsequent pairwise similarity comparison of the tumor cell clusters/groupings based on their phenotypic state using the Jaccard index to quantify similarities.

### Batch integration of snRNA-seq tumor cell data

To integrate the two different snRNA-seq profiles for annotated tumor cells only, we further filtered our expression datasets after our initial QC, described above, by applying the additional filters: (1) profiles must be associated with a singlet tumor cell based on DoubletDecon^46^ and inferCNV^11^, and (2) genes within each profile must have a minimum raw count of 2 in a minimum of 20 cells. The resulting datasets used for batch integration involved 3130 tumor cells expressing up to 11,298 genes (UW7 primary tumor cells) and 4388 PDX tumor cells expressing up to 7045 genes. Next, we utilized the suite of integration functions provided by Seurat v3.2.2 platform – *FindIntegrationAnchors* and *IntegrateData*. These functions apply canonical correlation analysis (CCA) to identify shared patterns in gene expression profiles between datasets, (i.e., “integration anchors” that are pairs of cells that share maximal correlation with one another). These anchors are then used as references with which the remaining datasets are harmonized with one another. The resulting integrated dataset consisted of 7277 tumor cells expressing up to 6807 genes.

### Batch integration of snRNA-seq and snATAC-seq data

To integrate snRNA-seq and snATAC-seq datasets, we utilized the suite of integration functions provided by the ArchR platform. Here, snATAC-seq data is first converted to gene score values, a correlate for gene expression, based on the accessibility of regulatory elements in the vicinity of a gene. Gene scores are determined via an exponential weighting function that takes into account ATAC-seq signals proximal to the transcription start site^18,47^. These values were calculated as part of the initial creation of ArrowFiles for each snATAC-seq dataset analyzed using the ArchR platform using the *CreateArrowFiles* function and default parameters. Alignment of snATAC-seq based gene scores to snRNA-seq expression data is performed using the *FindTransferAnchors* function from Seurat v3.2.2^18,29,47^.

### Quality control and snATAC-seq data preprocessing

Similar to snRNA-seq data, we initially processed 10X Genomics raw data using the Cell Ranger Software Suite (release 3.1.0). We performed additional data preprocessing and analysis using the software package ArchR (version 0.9.5). As part of the QC-filtering process, we used two metrics including: (1) number of unique nuclear fragments (>1000), and (2) signal-to-background ratio (i.e., transcription start site (TSS) enrichment score >4). This score represents a ratio of per-base pair accessibility centered around the TSS relative to flanking regions (2000 bp distal in either direction). Here, we used a TSS enrichment score value of 4 as a lower limit threshold. We also inspected fragment size distribution to verify whether a periodicity in fragment size, reflected as a multi-omic distribution, existed. These peaks and valleys in the distribution occur because fragments span 0, 1, 2, etc. nucleosomes and the Tn5 enzyme cannot cut DNA that is tightly wrapped around a nucleosome. We also inferred and removed those cells likely to be doublets from the datasets. Doublet inference in ArchR involves a method similar to the DoubletDecon in that heterotypic doublets were synthesized from the original population. These synthetic doublets were added to the original cell population and visualized via UMAP^48^. Single-cells were then labeled as putative doublets if they repeatedly projected as nearest neighbors during this iterative procedure.

We calculated QC statistics separately for each snATAC-seq dataset (UW7 primary tumor and UW7 recurrent tumor samples). The primary tumor set initially included 3770 cells, having a median of 31,462 fragments/cell. In this case, applying QC-filtering resulted in 3407 cells having a median of 29,268 fragments/cell. The recurrent tumor data set initially included 1934 single-cells, with a median of 8801 fragments/cell. Following QC-filtering, 1425 single-cells with a median of 8033 fragments/cell remained (Supplementary Figs. 15, 16).

### snATAC-seq dimensionality reduction

Due to the sparse nature of snATAC-seq data, popular methods like PCA would result in high inter-cell similarity due to the predominance of non-values in the snATAC-seq profiles across the single-cell samples. Towards addressing the sparsity issue, latent semantic indexing (LSI) was applied. LSI is a technique used in natural language processing to assess document similarity based on word counts, which often involves sparse and noisy datasets (many words, low frequency). Analogously, snATAC-seq profiles are viewed as a document and different accessible regions/peaks are words. To reduce the dimensionality of the snATAC-seq dataset, term frequency by depth normalization per cell was calculated. Next, these values were normalized by the inverse document frequency, which weights features by how often they occur. The result is a matrix that indicates how important a region/peak is to a sample. Using this resulting matrix, singular value decomposition (SVD) was applied to factorize the matrix into constituent matrices from which the most valuable information can be identified and projected into a lower dimensional space.

Here, ArchR applies a variation of this LSI methodology, an iterative LSI approach^49,50^. The default setting of two iterations was performed on both UW7 primary and matched recurrent tumor snATAC-seq datasets.

### snATAC-seq cell-type and transcriptional program labeling

Labeling of snATAC-seq datasets was performed using ArchR (package 22, v0.9.4). In brief, filtered fragments.tsv.gz files after quality control were used to generate an ArchR GeneScore matrix and a tiled genome feature matrix for each dataset. Cells were grouped by performing iterative latent semantic indexing (LSI) on the tile matrix, followed by the shared nearest neighbor clustering approach implemented in Seurat v3.2.2. GeneScore data, a correlate for gene expression, was then used to compare snATAC-seq clusters to a labeled reference snRNA-seq dataset, the UW7 primary tumor cells, using ArchR’s implementation of the *FindTransferAnchors* method from Seurat. Cell type and/or sample groups based on transcriptional network states with the highest score for each snATAC-seq cluster were used to annotate those cells for downstream analysis and visualization (Fig. 1c–i).

### Motif deviation scores

TF motif deviations were predicted on a per cell bases, relative to an aggregate background of a subpopulation of cells via chromVAR, which was incorporated into the broader ArchR package. The enrichment of TF motifs can guide in the prediction of which regulatory factors are most active in a cell type of particular interest, such as tumor cells. Designed for predicting enrichment of TF activity on a per-cell basis from sparse chromatin accessibility data, chromVAR produces two outputs including: (1) deviation – a TN5 insertion sequence bias-corrected measurement of how far the per-cell accessibility of a given motif deviates from the expected accessibility based on the average of all cells or samples, and (2) z-score – referred to as a “deviation score” for each bias-corrected deviation across all cells. The absolute value of the deviation score is correlated with the per-cell read depth. The greater the number of reads, the higher the confidence that the difference in per-cell accessibility of the given motif from the expectation is greater than that which would have occurred by chance.

### Regulatory network analysis

To infer regulons within single cells, we applied the SYGNAL^12^ and MINER^13^ workflow to the snRNA-seq dataset resulting from the Batch Integration procedure described above. The MINER algorithm involves a suite of functions that enables the inference of causal mechanistic relationships linking genetic mutations to transcriptional regulation. Because our datasets did not include any extensive mutational profiling, we primarily focused on identifying regulons, based on co-expression clustering and enrichment of transcription factor binding motifs present in those co-expressed genes, and calculated the activity of these regulons in the single-cell samples. Regulon activity represents the eigengene value in each single cell. Briefly, regulons are identified in part by PCA of snRNA-seq data profiles, i.e., PCA is used to identify PCs in which decreasing amounts of variation across genes is captured along each principal component – defined as a linear combination of samples in this approach. Here the coefficients, i.e., loadings, associated with each sample represent the eigengene value^51^. Alternatively, one can view eigengene values as a scalar representation of expression of gene members for a regulon. The eigenvalue represents a summarizing value of all the genes in the regulon and thus if these genes are indeed share co-regulation or are correlated, the eigengene value would be higher than that of randomly selected set of genes.

To determine the significance of each inferred regulon, we performed a permutation test to determine the possibility of obtaining an eigenvalue corresponding to the first principal component of a regulon (across all single-cells) of equal or greater value. Next, we randomly selected a set of genes having the same number of members as the original regulon and calculate the corresponding eigengene value for the permuted regulon. This procedure was repeated 1000 times to create a null distribution of eigengene values. We repeated this procedure for each inferred regulon. Those regulons whose eigengene values were greater than the 95th percentile of their respective null distribution were considered significant. Furthermore, we used eigenvalues to represent regulon “activity” within each cell.

Using the calculated activities of regulons, we identified groups of regulons sharing similar activity profiles across the cell population, i.e., transcriptional programs. Specifically, those regulons that correlated across the cell population (k-means clustering of sample pairwise Pearson correlations) defined distinct transcriptional programs. We further defined subpopulations of single-cells based on their shared regulon/transcriptional program activity. Sample pairwise Pearson correlations were calculated based on their regulon activity profiles.

### Regulon enrichment analysis

We used the gene set variance analysis GSVA (version 1.34.0, R package)^52^ to determine enrichment scores of gene sets. To confirm the significance of these enrichment scores, we performed permutation tests in which gene rankings were randomized in each single-cell sample and calculated the corresponding enrichment scores. In total, 1000 permutations were performed, from which the resulting scores were used to define empirically a null distribution of enrichment scores. We considered regulons having enrichment scores greater than the 95th percentile of the null distribution to be enriched in a particular cell.

### Projection of UW7 recurrent tumor cells onto UW7 primary and PDX tumor cell UMAP embeddings

Before projecting any new data onto some pre-existing latent space, we first determined a gene set shared across all datasets of interest. We identified 6,541 genes shared across the UW7 primary, PDX, and recurrent tumor snRNA-seq datasets. We performed PCA on the batch-integrated dataset (UW7 primary and PDX tumor cells) using only the 6,541 common set of genes and used the transformed gene expression data along the top 30 principal components for visualization via UMAP. Next, we mean-centered and variance-normalized the UW7 recurrent tumor cell expression data using gene-specific means and variances that were calculated from the batch-integrated dataset. These mean-centered and variance-normalized values were transformed via matrix multiplication with the eigenvectors from the top 30 PCs. We used the *predict* function in R along with the UMAP embeddings for the batch-integrated dataset to develop a linear regression model and the transformed UW7 recurrent tumor data (PC scores) as predictors. To determine which primary/PDX tumor cells were nearest neighbors to the projected recurrent tumor cells, we calculated pairwise Euclidean distances in the UMAP embedding space amongst all tumor cell pairs. Those cells having the lowest distance to the projected UW7 autopsy tumor cells were represented as arrowheads in Fig. 3b.

### Drug matching identification

To identify drugs targeting elements within the transcriptional programs and states identified from the network analysis, we applied the Open Targets platform tool (https://www.targetvalidation.org/). The platform integrates a variety of data and evidence from genetics, genomics, transcriptomics, drug, animal models, and literature to score and rank target-disease associations for drug target identification. We focused our search on identifying drug-target matches for only those drugs associated with any cancer treatment employed in any phase I–IV clinical trials.

### Reporting Summary

Further information on research design is available in the Nature Research Reporting Summary linked to this article.

## Supplementary information

Supplementary Information

REPORTING SUMMARY

Supplementary Tables 1-17

## Data Availability

Data generated for this study is available through the Gene Expression Omnibus (GEO) under the accession codes GSE189650 (UW7 snRNA-seq) and GSE157910 (UW7 scATAC-seq)^53^.

## Code availability

Relevant code is available upon request.
