# Supplementary material for: A single-cell based precision medicine approach using glioblastoma patient-specific models
Source: NPJ Precis Oncol. 2022 Aug 8;6:55. doi: 10.1038/s41698-022-00294-4 (PMC9360428; doi:10.1038/s41698-022-00294-4)
Supplement: Supplementary file 1 — Supplementary Information [file 41698_2022_294_MOESM1_ESM.docx]

**Supplementary Information**

**Title:**

A single-cell based precision medicine approach using glioblastoma patient-specific models

**Authors:**

James H. Park^1†^, Abdullah H. Feroze^2†^, Samuel N. Emerson^2†^, Anca B. Mihalas^2,3,^ C. Dirk Keene^4^, Patrick J. Cimino^4^, Adrian Lopez Garcia de Lomana^5^, Kavya Kannan^1^, Wei-Ju Wu^1^, Serdar Turkarslan^1^, Nitin S. Baliga1^6†*^, Anoop P. Patel^2,3,7†*^

**Affiliations:**

^1^ Institute for Systems Biology, Seattle, WA.

^2^ Department of Neurological Surgery, University of Washington, Seattle, WA.

^3^ Human Biology Division, Fred Hutchinson Cancer Research Center, Seattle, WA.

^4^ Department of Pathology, University of Washington, Seattle, WA.

^5^ Center for Systems Biology, University of Iceland, Reykjavik, Iceland.

^6^ Departments of Microbiology, Biology, and Molecular Engineering Sciences, University of Washington, Seattle, WA.

^7^ Brotman-Baty Institute for Precision Medicine, University of Washington, Seattle, WA.

† These authors contributed equally.

* Correspondence: nitin.baliga@isbscience.org, anoop.patel@duke.edu

**This PDF file includes:**

Supplementary Text

Supplementary References

Supplementary Figures 1 - 17 and corresponding legends

**Supplementary Text.**

***Identification of tumor cells based on inferred CNV state and gene marker expression.***

Copy number variation (CNV) states were inferred via inferCNV [1]. Briefly, snRNA-seq profiles were analyzed for evidence of large-scale chromosomal CNV including gains or deletions of large segments of a chromosome or entire chromosomes altogether. Expression intensity of genes across positions of the genome were compared to those in a reference set of cells, i.e., neurons identified based on expression levels of neuronal gene markers *RBFOX3* and *MAP2*. CNV states were estimated by sorting genes by their chromosomal location. A moving average was then calculated along each chromosome using a sliding window of 100 genes. Relative gene expression was capped to a minimum and maximum value of [-3, 3] to minimize the impact of any particular gene. Because GBM tumor cells exhibit a characteristic gain in Chr7 and loss in Chr10, we compared the CNV state of Chr7 and Chr10 genes across all cells relative to the previously identified internal reference set of neurons. Specifically, we calculated the sum of CNV states for Chr7 and Chr10 within each cell. We then defined a 95% confidence interval around the sample mean of the sum of gene expression for Chr7 or Chr10 across all reference cells using a student’s t-test distribution (ν = 197 – 1 degrees of freedom, based on the number of reference neurons identified). Those cells simultaneously having a sum greater than the 95% confidence interval range for Chr7 and less than the 95% confidence interval range for Chr10 were annotated as potential GBM tumor cells.

We then mapped the preliminary CNV-based tumor-cell annotation onto UMAP embeddings of the processed snRNA-seq profiles and compared the preliminary annotation against additional quantitative metrics including: 1) expression levels for GBM-associated gene markers *EGFR*, *SOX9*, and *PTPRZ1*, and 2) relative proximity of tumor and non-tumor cells to one another in the UMAP space, i.e., relative clustering of potential tumor cells. Using a combination of these metrics, we refined the preliminary cell annotation to define clusters of putative tumor cells (Figs. S1, S12).

***Projection of UW7 recurrent autopsy samples onto UW7 primary and PDX tumor cells.***

To project new data onto a pre-existing UMAP space, a common set of gene features is required. Comparison of the final gene sets, following QC filtering, across the UW7 primary tumor, UW7 PDX samples, and UW7 recurrent tumor biopsy revealed 6,541 genes common across the three snRNA-seq datasets. We then reprocessed the batch-integrated primary and PDX tumor cells, based on the 6,541 common gene set, following a workflow similar to that described in the Methods. First, we performed PCA on the refined batch-integrated (primary and PDX tumor cell snRNA-seq) dataset. Second, we visualized the data by applying UMAP on the rotated dataset, i.e., scores along the first 30 PCs. This defined the UMAP space onto which the recurrent tumor cells would be co-embedded.

Next, we projected recurrent tumor cells into the PC space defined by the batch-integrated snRNA-seq dataset (primary and PDX tumor cells) to determine the rotations (scores) of the recurrent tumor cells that would be used for UMAP embedding. We mean-centered and scaled the normalized recurrent tumor cell snRNA-seq profiles, on a gene-centric basis, using the means and standard deviations defined by the batch-integrated primary and PDX tumor cell dataset. We then projected a recurrent tumor cell onto the pre-existing PCA space by calculating the dot product between its mean-centered and scaled gene expression profile and the eigenvector defining a particular principal component. The resulting scalar product represented the score value of that recurrent tumor cell along that particular principal component. Finally, the score values for the projected recurrent tumor cells along the first 30 PCs were embedded into the UMAP space using the linear regression function *lm* in R.

***ArchR quality control metrics of snATAC-seq data.***

We followed the guidelines outlined in the ArchR package [2] to assess the quality of the snATAC-seq data obtained from UW7 primary and matched recurrent tumor biopsies. Per the ArchR platform, three metrics are used to assess quality of snATAC-seq samples: 1) number of unique fragments (not mapping to mitochondrial DNA) per cell, 2) signal-to-background ratio, i.e., transcription start site (TSS) enrichment score, and 3) fragment size distribution based on nucleosomal periodicity. Below we briefly summarize the reasoning behind each QC metric, which is described in greater detail by Granja *et al*. [3].

The number of unique fragments as QC metric enables the identification of cells that have a low number of unique fragments, which provided a metric to filter out cells that would not contribute any meaningful information on chromatin accessibility states. TSS enrichment score is based on the idea that ATAC-seq data is universally enriched at gene TSS regions as compared to other genomic regions due to large protein complexes that bind to promoters. By looking at per-bp accessibility centered at these TSS regions, we see a local enrichment relative to the surrounding regions (1900-2000 bp distal in both directions). The ratio between the peak of this enrichment (centered at the TSS) relative to these surrounding regions represents the TSS enrichment score.

Fragment size distribution provides yet another method to assess quality, providing a way to perform a sanity-check on data quality. DNA wraps around nucleosomes in a patterned way, where ~147 bp of DNA wrap tightly around a nucleosome. These tightly wrapped DNA regions cannot be cut by the Tn5 transposase. Consequently, fragment size distributions will often be depleted at values that are of the length of DNA wrapped around a nucleosome. A result of this patterned DNA wrapping around a nucleosome is a periodicity in the distribution of fragment sizes in the data. Thus, the size distribution will likely consist of hills and valleys because fragments must span 0, 1, 2, etc. nucleosomes.

***Differential expression analysis of UW7 primary tumor and PDX single cells***

We analyzed the UW7 primary and PDX tumor cell snRNA-seq profiles independently to identify common/distinct phenotypic behaviors between the sample sets. To that end, we performed a combination of unsupervised, shared nearest neighbor (SNN) clustering, differential expression analysis [4], and enrichment analyses.

The primary tumor cells, which organized into 11 cell clusters, expressed 3,531 differentially expressed genes (DEGs) (Supplementary Table 10). Enrichment analysis revealed that multiple functional gene sets [5–7] associated with established GBM-processes were enriched in the upregulated DEGs of the primary tumor SNN-clusters (Supplementary Table 11). For example, genes associated with hypoxia, glycosylation, and epithelial-to-mesenchymal transition were enriched in primary tumor cluster 0 (Supplementary Fig. 5). Separately, angiogenesis genes were enriched only in primary tumor cell cluster 5, while significant enrichment of gene sets associated with cell-cycle functions was found only in SNN-cluster 11, suggesting that this particular cell cluster consisted primarily of proliferating tumor cells. In addition, primary tumor cells in SNN-cluster 8 were enriched with MYC targets genes, which have been shown to have clinical prognostic value [8] as well as classification utility in IDH-wild type GBMs [9]. Furthermore, both classical (CL) and proneural (PN) gene sets [10] were enriched across multiple SNN-clusters; clusters 1 and 7 were enriched for CL subtype genes, clusters 5 and 11 were enriched for PN subtype genes, and cluster 4 was enriched for both PN and CL genes (Supplementary Table 11).

Concomitantly, PDX tumor cells organized into seven SNN-clusters (Fig. 2D), expressing 1,731 DEGs (Supplementary Table 12). Enrichment analysis of the upregulated DEGs associated with the seven SNN-clusters revealed multiple enriched processes in primary tumor cell SNN-clusters including: hypoxia and epithelial-mesenchymal transition (EMT) (PDX SNN-cluster 5, primary tumor SNN-cluster 0); cell-cycle-related functions including E2F targets and G2M checkpoints (PDX SNN-cluster 0, primary tumor SNN-cluster 11); and UV response down (PDX SNN-clusters 2, 4, 5, and primary tumor SNN-cluster 5). Multiple GBM subtype genes were also enriched across the PDX tumor cell SNN-clusters. Classical subtype genes were enriched in PDX tumor cell clusters 0, 1, and 4, while PN subtype genes were enriched in PDX tumor cell SNN-clusters 2, and 6 (Supplementary Table 13). Conversely, several PDX SNN-clusters, which were composed primarily of SOC-treated tumor cells, were enriched for genes and corresponding pathways (e.g., KRAS signaling pathway, Supplementary Table 13), not enriched in any other PDX or primary tumor cell SNN-clusters. These similarities and differences (Supplementary Fig.

6), in addition to those outlined in the main text, highlight the ability of the PDX models to recapitulate phenotypes observed in the primary tumor as well unique phenotypes induced by SOC-treatment.

***Batch integration, data integrity, and network-inference accuracy***

Typically, batch integration methods for single-cell RNA-seq data are used for visualization. However, a potential concern with their use is the introduction of technical artifacts into the resulting batch-integrated data set. Ideally, when integrating multiple single-cell batches, a subset of samples across the batches would be from identical conditions or cell type. This common subset of single cells would be used for alignment and subsequent integration. Given the pervasive heterogeneity of GBM tumors and the uncertainty of having multiple subpopulations of similar tumor cells implanted into multiple PDX mice, there was a higher chance for dissimilar subpopulations being used for batch alignment, which would result in technical artifacts negatively affecting the data. To address this concern, we concomitantly performed network inference analyses of the integrated-, primary-, and PDX-datasets and compared the inferred regulons and transcriptional programs to one another using multiple quantitative analyses including: 1) semantic similarity analysis of gene ontology terms [11,12] across all regulons, 2) pairwise comparison of all transcriptional states based on their phenotypic state (Methods), and 3) statistical enrichment of overlapping genes across transcriptional programs (Methods, Fig S8). Results indicated that multiple regulons and transcriptional programs across the integrated-, tumor-, and PDX-datasets were similar to one another.

Finally, we compared the (integrated) transcriptional states of primary tumor cells to those of the (non-integrated) untreated PDX cells that were clustered together in the UMAP co-embedding space (Fig 3A, Fig S9) using cosine similarity. Pairwise comparison of transcriptional states between parental tumor cells and untreated PDX samples within the same SNN-cluster resulted in distributions of cosine similarity values that were: 1) equivalent to the distribution of cosine similarity values determined from pairwise comparison of primary tumor cells (amongst each other) within the same SNN-cluster, 2) significantly greater than the cosine similarity distribution of primary tumor cells compared to untreated (non-integrated) PDX samples not belonging to the SNN-cluster of interest, and 3) greater than the cosine similarity distribution determined from randomly permutated gene expression (and consequently transcriptional states) of tumor cells within the SNN-cluster of interest (Supplementary Fig. 10). Together, these results indicate that the batch integration step did not severely affect the results from the network-inference or subsequent downstream analyses.

***Subpopulation of integrated primary and PDX tumor cells***

Following batch integration of the primary and PDX tumor, dimensionality reduction via principal component analysis (PCA) and visualization via UMAP, we observed two broad cell groupings (Supplementary Fig. 9). Subsequent downstream analysis, focused on the larger cluster of cells, which consisted of 6,305 cells (86.6% of all primary and PDX tumor cells), from which the longitudinal stages were derived. The second, smaller cluster of cells, labeled as “subpopulation”, consisted of 972 cells (13.4% of all primary and PDX tumor cells). The majority of subpopulation cells were from the primary tumor (95.4%). These cells was enriched with primary tumor cells belonging to SNN-clusters 0, 3, 6, 8 (Fig. 2A) and PDX tumor cells belonging to PDX tumor-cell SNN-cluster 2 (Fig. 2B). A large majority of the primary tumor cells making up the subpopulation set originated from primary tumor SNN-cluster 3 (414 cells, 42.6% of the primary tumor cells in this set), followed by primary tumor-cell SNN-cluster 6 (268 cells, 27.5%), and SNN-cluster 8 (176 cells, 18.1%). Primary tumor-cell SNN-cluster 3 was enriched with a wide range of gene sets related to hypoxia, hemostasis, WNT signaling, epithelial-to-mesenchymal transition, and adult tissue stem modules, among others (Supplementary Table 11). Similarly, primary tumor SNN-clusters 6, and 8, which were phenotypically similar to each other, were enriched for gene sets associated with tumor invasiveness, stem-cell-related genes, and translation initiation among others (Supplementary Fig. 9, Supplementary Table 11). Only a small number of PDX SNN-cluster 2 tumor cells (33 out of 671 tumor cells) were grouped into the subpopulation set of cells.

***Multiple regulons regulated by identical TF***

When assessing potential targets, it is critical to consider that multiple regulons may be controlled by a single master regulator yet have activities that are contextually disparate. One example involves the androgen receptor (*AR*), which putatively regulates three regulons (Supplementary Supplementary Table 3, Supplementary Fig. 17). Regulon-15 demonstrated “selected against” behavior, suggesting that SOC was effective at suppressing its activity. Regulon-20 demonstrated “transient” behavior, suggesting that it may have some activity during SOC and may relate to resistance to radiation [13]. Finally, regulon-17 demonstrated “induced” behavior and had high activity at recurrence. Activity of the AR regulon raises the possibility of androgen targeting therapies as possible treatments for GBM in certain contexts, including its concurrent use with SOC therapy or in the adjuvant and salvage settings. Analysis of additional samples is required to determine whether this is a gender-specific effect (this patient was male) and whether *AR* activity is generalizable across other patients will require analysis of additional samples.

**Supplementary References**

1. Tickle, T.; Tirosh, I.; Georgescu, C.; Brown, M.; Haas, B. inferCNV of the Trinity CTAT Project. 2019.

2. Granja, J.; Corces, R. ArchR Project Available online: https://www.archrproject.com/articles/Articles/tutorial.html.

3. Granja, J.M.; Corces, M.R.; Pierce, S.E.; Bagdatli, S.T.; Choudhry, H.; Chang, H.Y.; Greenleaf, W.J. ArchR: An integrative and scalable software package for single-cell chromatin accessibility analysis. *bioRxiv* **2020**, doi:10.1101/2020.04.28.066498.

4. Stuart, T.; Butler, A.; Hoffman, P.; Hafemeister, C.; Papalexi, E.; Mauck, W.M.; Hao, Y.; Stoeckius, M.; Smibert, P.; Satija, R. Comprehensive Integration of Single-Cell Data. *Cell* **2019**, *177*, 1888-1902.e21, doi:https://doi.org/10.1016/j.cell.2019.05.031.

5. Subramanian, A.; Tamayo, P.; Mootha, V.K.; Mukherjee, S.; Ebert, B.L.; Gillette, M.A.; Paulovich, A.; Pomeroy, S.L.; Golub, T.R.; Lander, E.S.; et al. Gene set enrichment analysis: A knowledge-based approach for interpreting genome-wide expression profiles. *Proc. Natl. Acad. Sci.* **2005**, *102*, 15545–15550, doi:10.1073/pnas.0506580102.

6. Liberzon, A.; Subramanian, A.; Pinchback, R.; Thorvaldsdóttir, H.; Tamayo, P.; Mesirov, J.P. Molecular signatures database (MSigDB) 3.0. *Bioinformatics* **2011**, *27*, 1739–1740, doi:10.1093/bioinformatics/btr260.

7. Liberzon, A.; Birger, C.; Thorvaldsdóttir, H.; Ghandi, M.; Mesirov, J.P.; Tamayo, P. The Molecular Signatures Database (MSigDB) hallmark gene set collection. *Cell Syst.* **2015**, *1*, 417–425, doi:10.1016/j.cels.2015.12.004.

8. Cho, S.Y.; Kim, S.; Kim, G.; Singh, P.; Kim, D.W. Integrative analysis of KIF4A, 9, 18A, and 23 and their clinical significance in low-grade glioma and glioblastoma. *Sci. Rep.* **2019**, *9*, 4599, doi:10.1038/s41598-018-37622-3.

9. Chang, Y.-Z.; Li, G.-Z.; Pang, B.; Zhang, K.-N.; Zhang, X.-H.; Wang, Y.-Z.; Jiang, Z.; Chai, R.-C. Transcriptional Characteristics of IDH-Wild Type Glioma Subgroups Highlight the Biological Processes Underlying Heterogeneity of IDH-Wild Type WHO Grade IV Gliomas. *Front. Cell Dev. Biol.* **2020**, *8*, 1132, doi:10.3389/fcell.2020.580464.

10. Verhaak, R.G.W.; Hoadley, K.A.; Purdom, E.; Wang, V.; Qi, Y.; Wilkerson, M.D.; Miller, C.R.; Ding, L.; Golub, T.; Mesirov, J.P.; et al. Integrated Genomic Analysis Identifies Clinically Relevant Subtypes of Glioblastoma Characterized by Abnormalities in PDGFRA, IDH1, EGFR, and NF1. *Cancer Cell* **2010**, *17*, 98–110, doi:10.1016/j.ccr.2009.12.020.

11. Yu, G. Gene Ontology Semantic Similarity Analysis Using GOSemSim. *Methods Mol. Biol.* **2020**, *2117*, 207–215, doi:10.1007/978-1-0716-0301-7_11.

12. Yu, G.; Wang, L.-G.; Han, Y.; He, Q.-Y. clusterProfiler: an R Package for Comparing Biological Themes Among Gene Clusters. *Omi. A J. Integr. Biol.* **2012**, *16*, 284–287, doi:10.1089/omi.2011.0118.

13. Werner, C.K.; Nna, U.J.; Sun, H.; Wilder-Romans, K.; Dresser, J.; Kothari, A.U.; Zhou, W.; Yao, Y.; Rao, A.; Stallard, S.; et al. Expression of the Androgen Receptor Governs Radiation Resistance in a Subset of Glioblastomas Vulnerable to Antiandrogen Therapy. *Mol. Cancer Ther.* **2020**, *19*, 2163–2174, doi:10.1158/1535-7163.MCT-20-0095.

14. Neftel, C.; Laffy, J.; Filbin, M.G.; Hara, T.; Shore, M.E.; Rahme, G.J.; Richman, A.R.; Silverbush, D.; Shaw, M.L.; Hebert, C.M.; et al. An Integrative Model of Cellular States, Plasticity, and Genetics for Glioblastoma. *Cell* **2019**, doi:https://doi.org/10.1016/j.cell.2019.06.024.

15. Wang, L.; Babikir, H.; Müller, S.; Yagnik, G.; Shamardani, K.; Catalan, F.; Kohanbash, G.; Alvarado, B.; Di Lullo, E.; Kriegstein, A.; et al. The phenotypes of proliferating glioblastoma cells reside on a single axis of variation. *Cancer Discov.* **2019**, *9*, 1708–1719, doi:10.1158/2159-8290.CD-19-0329.

**Supplemental Figures and Legends**

**Supplementary Figure 1.**

**
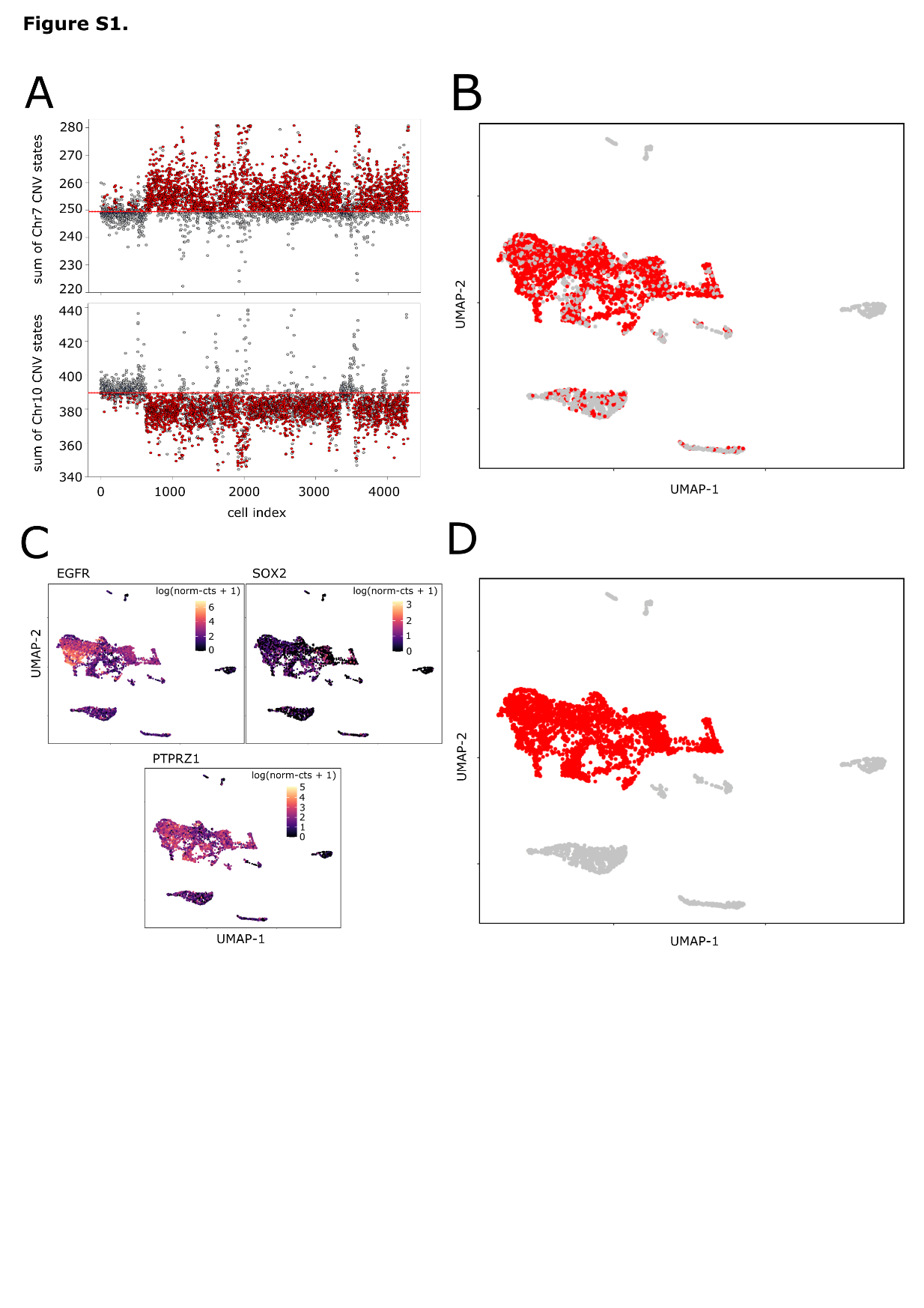
**

**Supplementary Figure 1. Tumor cell annotation in primary tumor biopsy.** **(A)** Sum of inferred copy number variation (CNV) states for Chr7 and Chr10 across 4,183 cells collected from primary tumor biopsy. Red solid lines represent the mean sum of inferred CNV state while dashed lines represent a 95% confidence interval of the average sum of inferred CNV state for reference neurons, classified as such per expression of neuronal markers RBFOX3 and MAP2. Red points represent cells having a Chr7 and Chr10 CNV state above and below, respectively, the confidence-level threshold based on CNV states of the reference cells. **(B)** UMAP plot of UW7 primary tumor snRNA-seq profiles. Red colored cells represent cells having an inferred CNV gain in Chr7 and loss in Chr10, shown in (A). **(C)** UMAP plot of UW7 primary tumor biopsy snRNA-seq profiles annotated according to log-normalized gene counts of tumor gene markers EGFR, SOX2, and PTPRZ1. **(D)** Final tumor-cell annotation of UW7 primary tumor cell. Based on inferred CNV state and the expression of tumor gene markers, 3,130 cells of the total 4,183 cells analyzed from the parental tumor biopsy were defined as tumor cells.

**Supplementary** **Figure 2.**

**
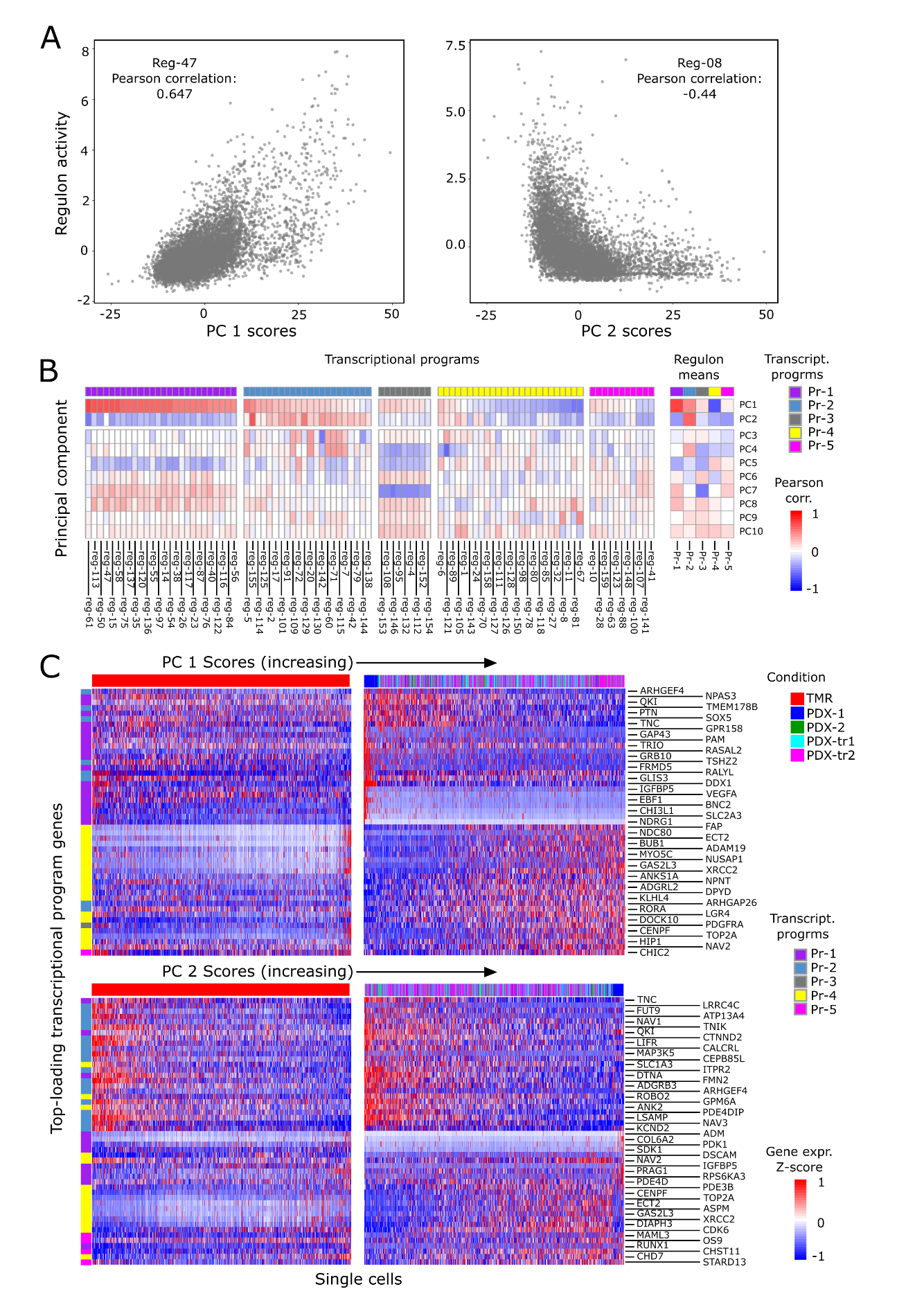
**

**Supplementary Figure 2. Transcriptional program activities correlate with PC scores.** To determine whether transcriptional programs identified incorporate highly varying genes, we examined how transcriptional program activity levels within cells correlated with their corresponding PC sample scores. **(A)** Scatter plots of regulon activity vs. PC scores across primary and PDX tumor cells. In many cases, activities of significant regulons (Supplementary Table 3), positively or negatively correlated with PC scores like regulon-47 or regulon-8, respectively. **(B)** Heatmap of pairwise Pearson correlation of regulon activities and scores along PCs (rows) across UW7 primary tumor cells. Top color bar indicates the transcriptional program to which a particular regulon (column) belongs. Regulons and transcriptional programs do co-vary with scores along PCs 1-10. **(C)** Heatmaps of the expression of top-loading genes (based on absolute loading values) within transcriptional programs across primary (left) and PDX (right) tumor cells. Samples (columns) are rank ordered according to their sample score along PC 1 (top row) and PC 2 (bottom row). Relative gene expression depicted are (non-integrated) primary and PDX tumor snRNA-seq data. Left-adjacent color bars on heatmaps indicate transcriptional programs to which an individual gene belongs.

**Supplementary Figure 3.**


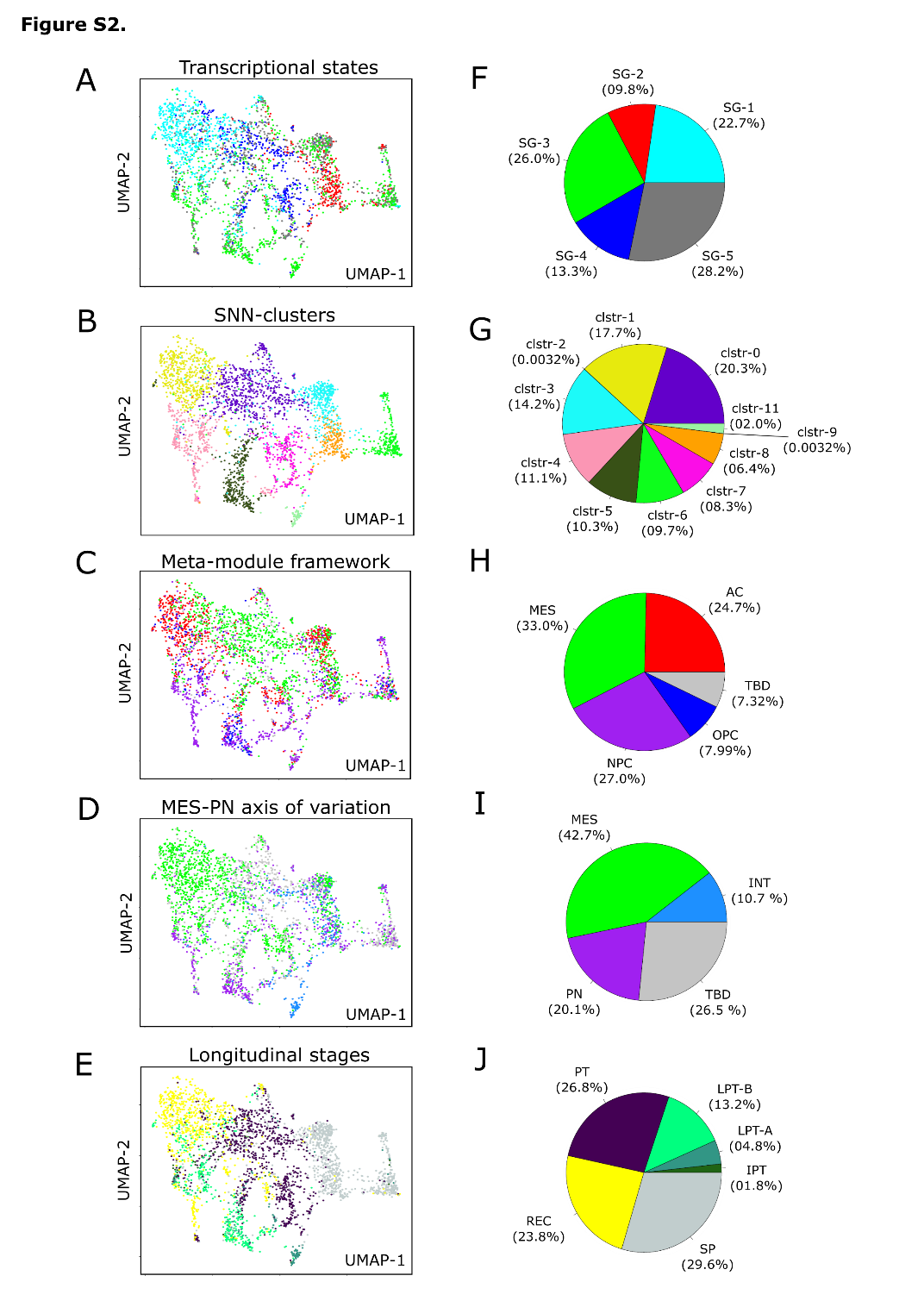


**Supplementary Figure 3. Cell type annotation of UW7 primary tumor cells.** UMAP plots of non-integrated snRNA-seq profiles of UW7 primary tumor cells annotated according to **(A)** transcriptional network states, **(B)** shared nearest neighbor clusters , **(C)** meta-module cell states [14], **(D)** MES-PN axis of differentiation [15], and **(E)** Longitudinal stages (Fig 3B). Cells unable to be categorized into any of the preexisting cell state frameworks [14,15] were labeled as TBD. **(F-J)** Breakdown of the UW7 primary tumor population for each corresponding annotation framework. Clstr – cluster, AC – astrocyte, MES – mesenchymal, NPC – neural progenitor cell, TBD – unclassified, PT – pretreatment, IPT – immediate post-treatment, LPT-A – late stage post-treatment A, LPT-B – late stage post-treatment B, REC – recurrent, SP – subpopulation of cells that did not fall into corresponding longitudinal stages (Supplemental text).

**Supplementary Figure 4.**

**
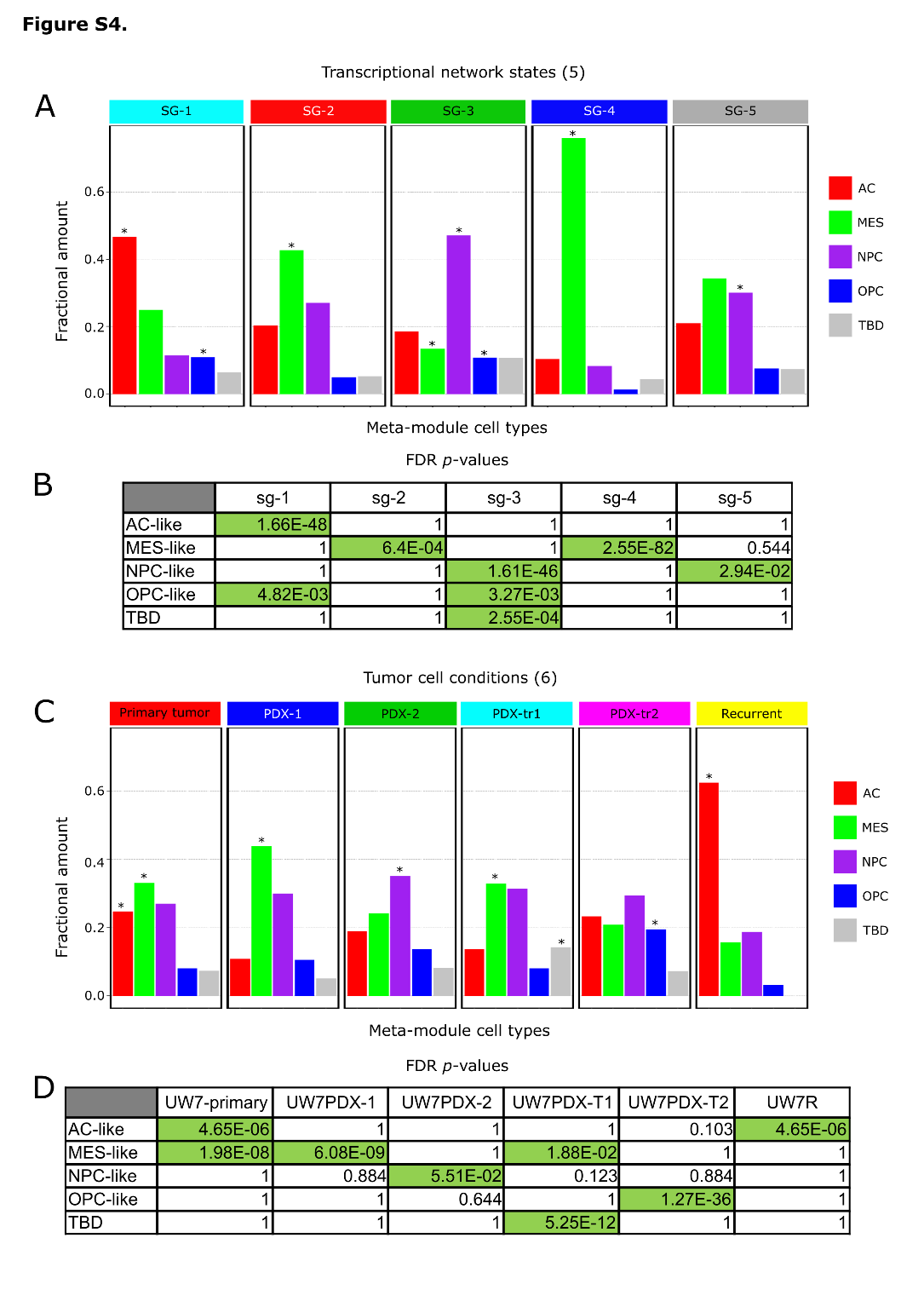
**

**Supplementary Figure 4. Enrichment of meta-module cell states in transcriptional network states.** **(A)** Proportion of cells assigned to a meta-module cell state comprising a transcriptional network state. Asterisks indicate meta-module cell state(s) significantly enriched within each network state. **(B)** Breakdown of enrichment significance (FDR *p*-value) for meta-module cell states within network states. Green-colored cells in table indicate FDR *p*-values << 0.01. **(C)** Proportion of meta-module cell states comprising the total amount of primary, PDX, and recurrent tumor cells (collected at autopsy). Asterisks indicate meta-module cell state(s) significantly enriched within primary, PDX, and recurrent tumor cells. **(D)** Breakdown of enrichment (FDR *p*-values) for meta-module cell states within PDX treatment conditions. Green-colored cells in table indicate FDR *p*-values << 0.01.

**Supplementary Figure 5.**

**
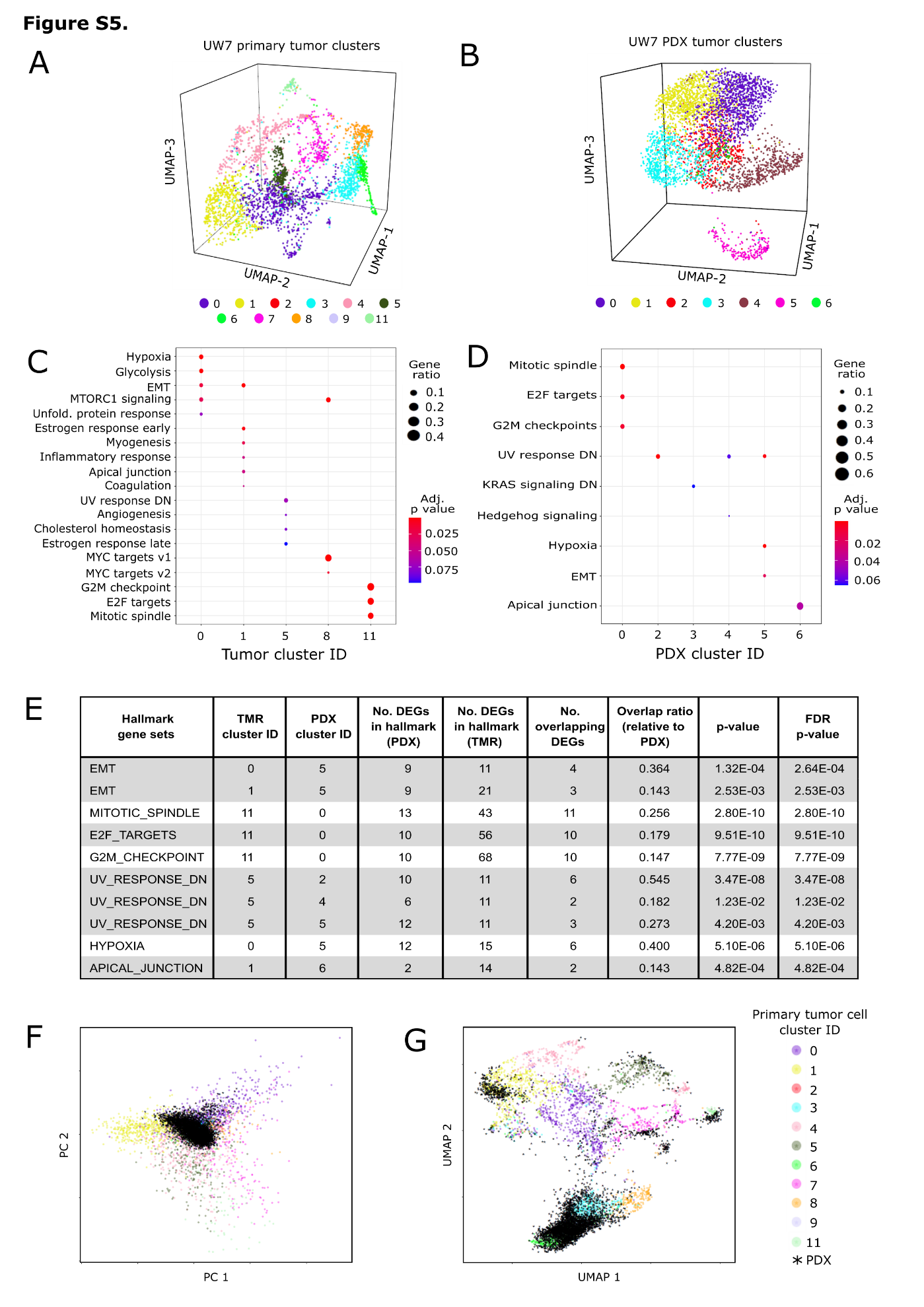
**

**Supplementary Figure 5. PDX (untreated) samples exhibit similar transcriptional-program gene expression profiles with UW7 primary tumor cells.** UMAP plots from Fig. 2 visualizing high-dimensional snRNA-seq profiles of **(A)** UW7 primary and **(B)** UW7 PDX tumor cells, for reference. **(C)** Dot plot of hallmark gene sets enriched in upregulated DEGs associated with primary tumor-cell SNN-clusters. Node size indicates the ratio of upregulated DEGs belonging to a particular hallmark gene sets. Color indicates significance level of enrichment (FDR-adjusted p-value). **(D)** Dot plot of hallmark gene sets enriched in upregulated DEGs associated with PDX tumor-cell SNN-clusters. **(E)** Table summarizing number of overlapping genes (and enrichment) shared between primary and PDX tumor-cell SNN-clusters for a specific hallmark function. **(F)** PCA of primary tumor single-cell samples. PDX samples (*) have been projected onto the PC1 vs PC2 scores space defined by the primary tumor cells (Methods). **(G)** UMAP plot shows embeddings of primary tumor samples (Methods). PDX samples (*) have been co-embedded onto the same latent space defined by primary tumor cells.

**Supplementary Figure 6.**

**
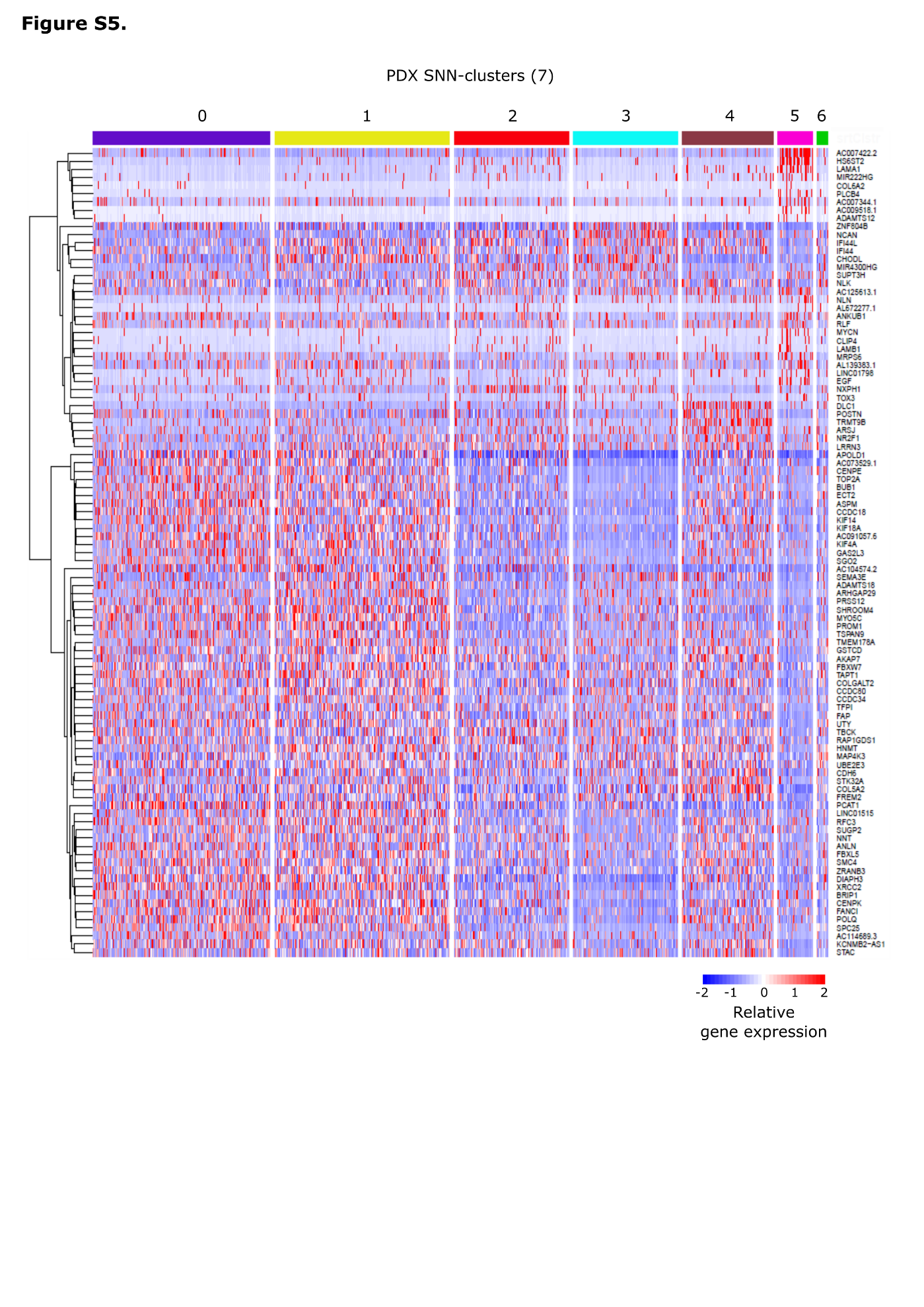
**

**Supplementary Figure 6. DEGs unique to PDX tumor cells.** Heatmap of normalized gene expression (Methods) of DEGs unique to PDX tumor cells. Genes (rows) were hierarchically clustered. Single-cell samples (columns) are grouped based on SNN-cluster membership (Fig 2D).

**Supplementary Figure 7.**

**
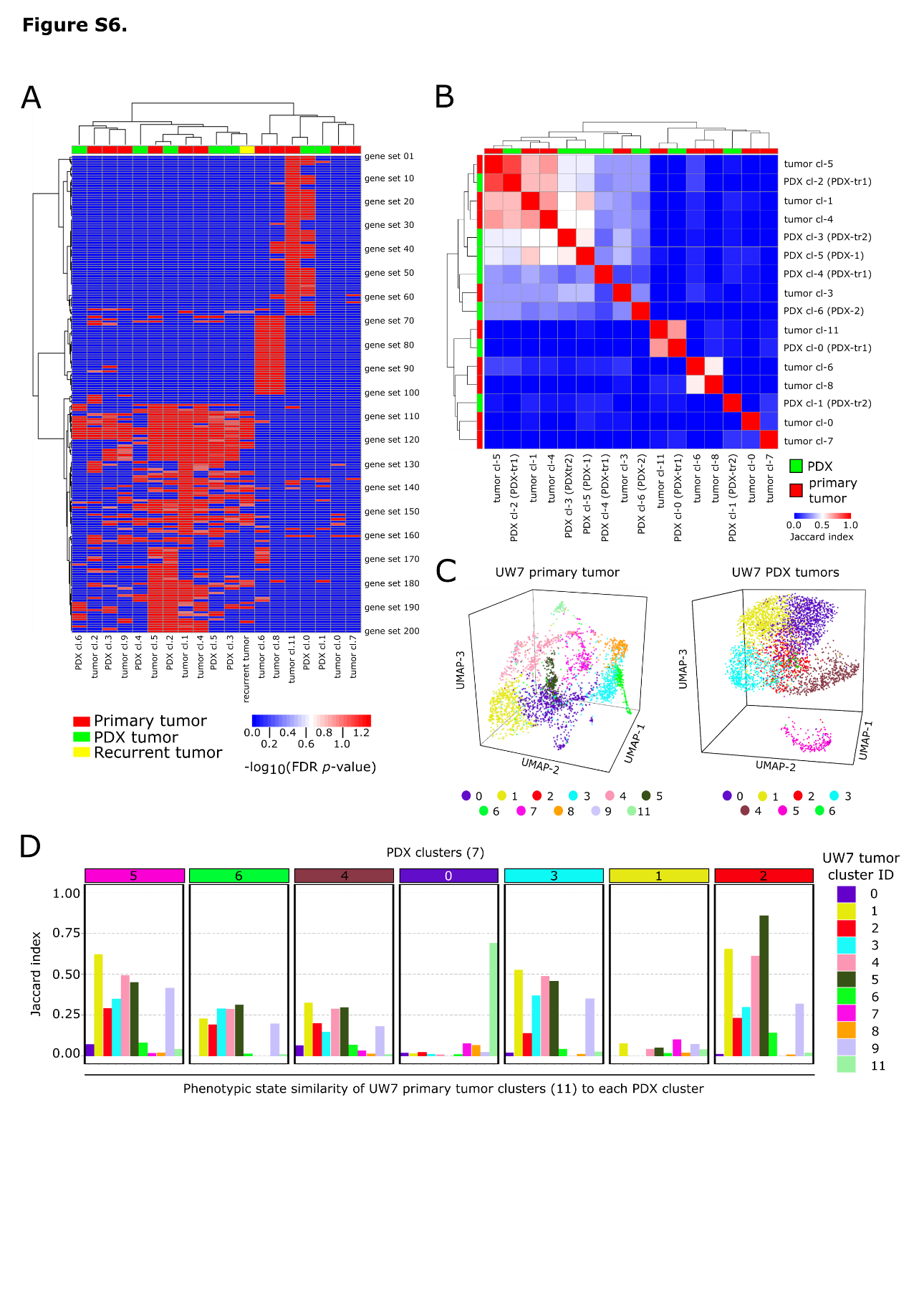
**

**Supplementary Figure. 7. Phenotypic comparison of UW7 primary, PDX, and recurrent tumor-cell SNN clusters.** **(A)** Heatmap of significance values (-log_10_(FDR-adjusted *p*-values)) of top 200 enriched gene sets (Methods). Aggregation of significant functional gene sets (-log_10_(FDR-adjusted *p*-value) ≥ 1) defines the phenotypic state of cells belonging to an SNN-cluster of interest. MSigDB gene set comprising the phenotypic states are described in Supplementary Table 14. **(B)** Heatmap of Jaccard indices determined from pairwise comparison of phenotypic states (Methods) of primary and PDX tumor-cell SNN-clusters. **(C)** UMAP plots of primary and PDX tumor cells, identical to those in Fig. 2A-B, are included for visual reference. **(D)** Jaccard indices from pairwise comparison of primary tumor-cell SNN-clusters to respective PDX tumor-cell SNN-cluster, labeled above each bar plot.

**Supplementary Figure 8.**

**
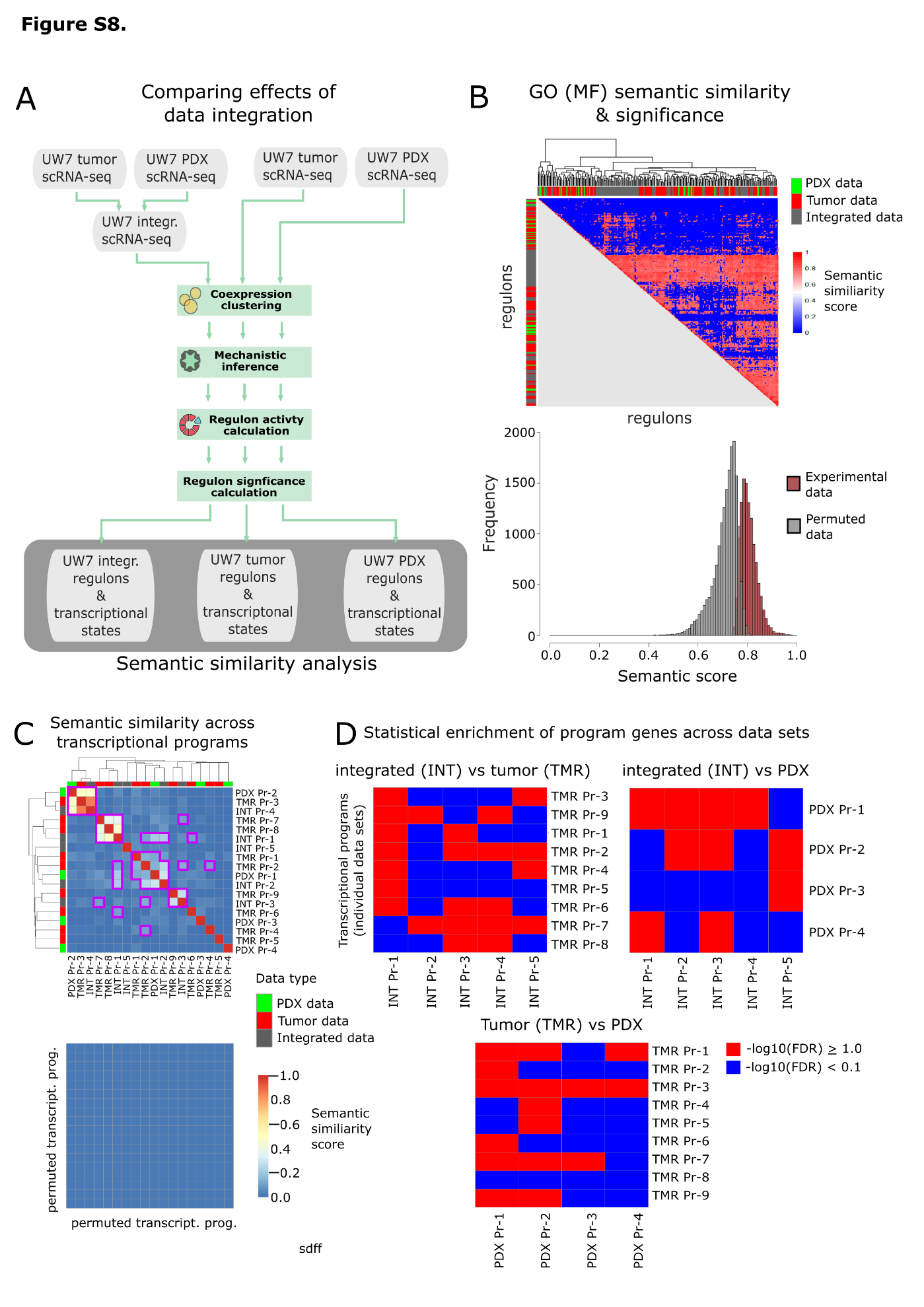
**

**Supplementary Figure 8. Regulon and transcriptional program similarities.** **(A)** Flow diagram of network inference analyses performed on the UW7 primary and UW7 PDX tumor data. **(B)** Heatmap of symmetric matrix indicating significance of semantic similarity scores based on GO::MF terms across all pairwise comparisons of regulons derived from the integrated and non-integrated primary and PDX tumor snRNA-seq data. The lower triangular region of the heatmap is grayed out in the symmetric matrix. Pixel color indicate significant (red) or non-significant (blue) semantic similarity scores. Color bars indicate the data source (integrated data – gray, primary tumor data – red, PDX tumor data – green). Dendrogram represents hierarchical clustering of regulons based on their pairwise semantic similarity scores. Histogram of significant (red) and non-significant (gray) semantic similarity scores as determined by permutation analysis (see Methods). Semantic similarity scores were determined using the clusterProfiler package in R. **(C)** Heatmap (left) of semantic similarity scores of transcriptional programs from network inference analysis. Left adjacent dendrogram represents clustering of programs based on pairwise semantic similarity scores. Color bars along the left and top of the heatmap indicate the data source from which a transcriptional program was derived (color code identical to that in subpanel (B)). Heatmap (right) of semantic similarity scores derived from permutation test, in which an equal number of genes for each transcriptional program was selected and a subsequent functional enrichment analysis via clusterProfiler was performed, followed by semantic similarity analysis (repeated 1000 iterations). **(D)** Heatmaps of statistical enrichment of genes from programs originally identified from integrated snRNA-seq data set in those programs identified from the analysis of the non-integrated primary tumor and PDX data sets, respectively. Red pixels indicate significant enrichment, (–*log_10_(FDR-adjusted p values) ≥ 1.0* ), while blue pixels indicate no enrichment *(–log_10_(FDR adjusted p values) < 1.0)*.

**Supplementary Figure 9.**


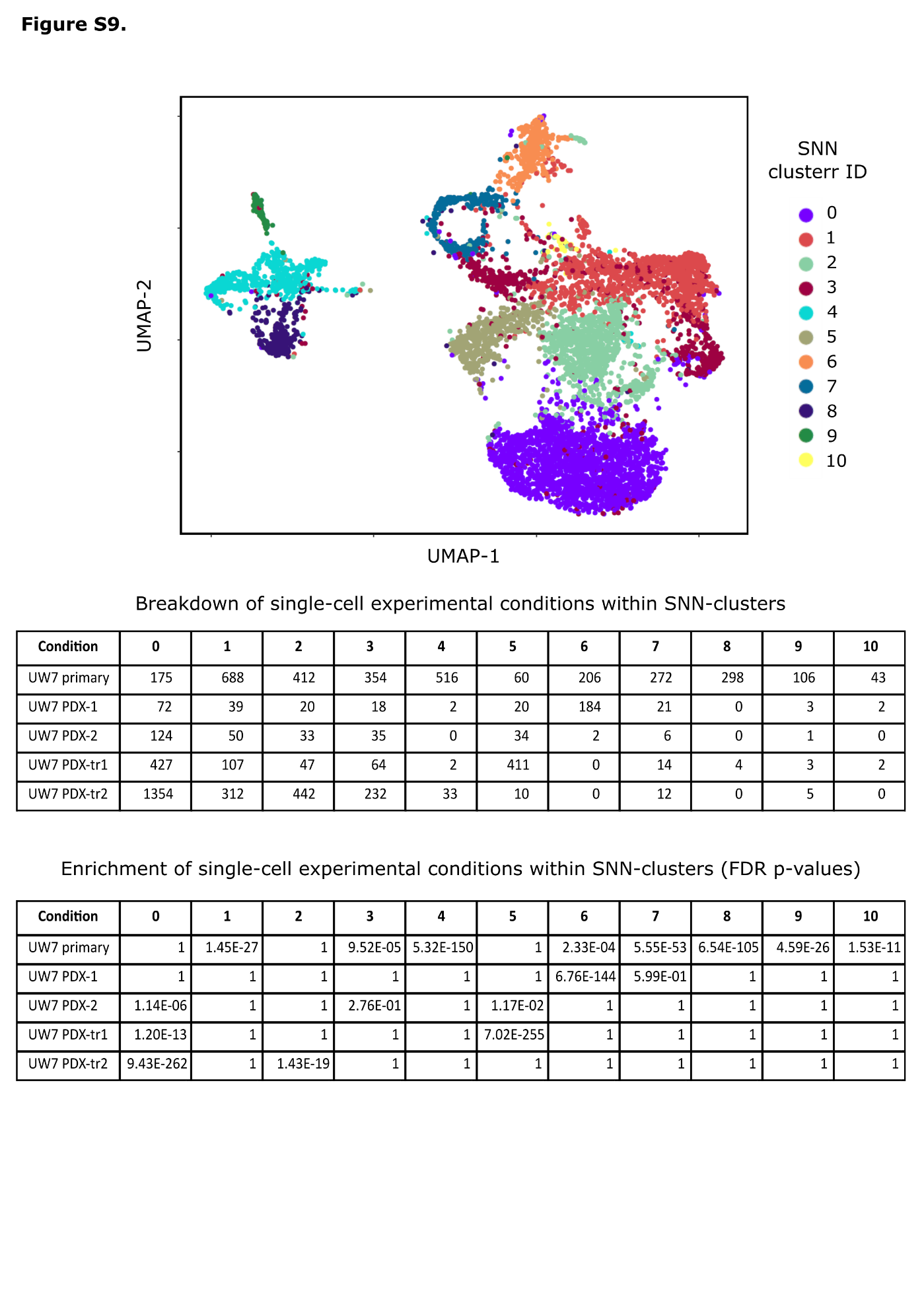


**Supplementary Figure 9.** **Enrichment of tumor sample conditions within shared-nearest-neighbors (SNN) clusters of batch-integrated primary tumor and PDX snRNA-seq data.** Single-cell clusters identified using SNN modularity optimization and Leiden algorithm using the Seurat v3.2.2 platform. Top table tabulates composition of tumor/treatment conditions within each SNN-cluster. Bottom table tabulates FDR-adjusted *p*-values for enrichment of experimental conditions within each cluster, per hypergeometric test.

**Supplementary Figure 10.**


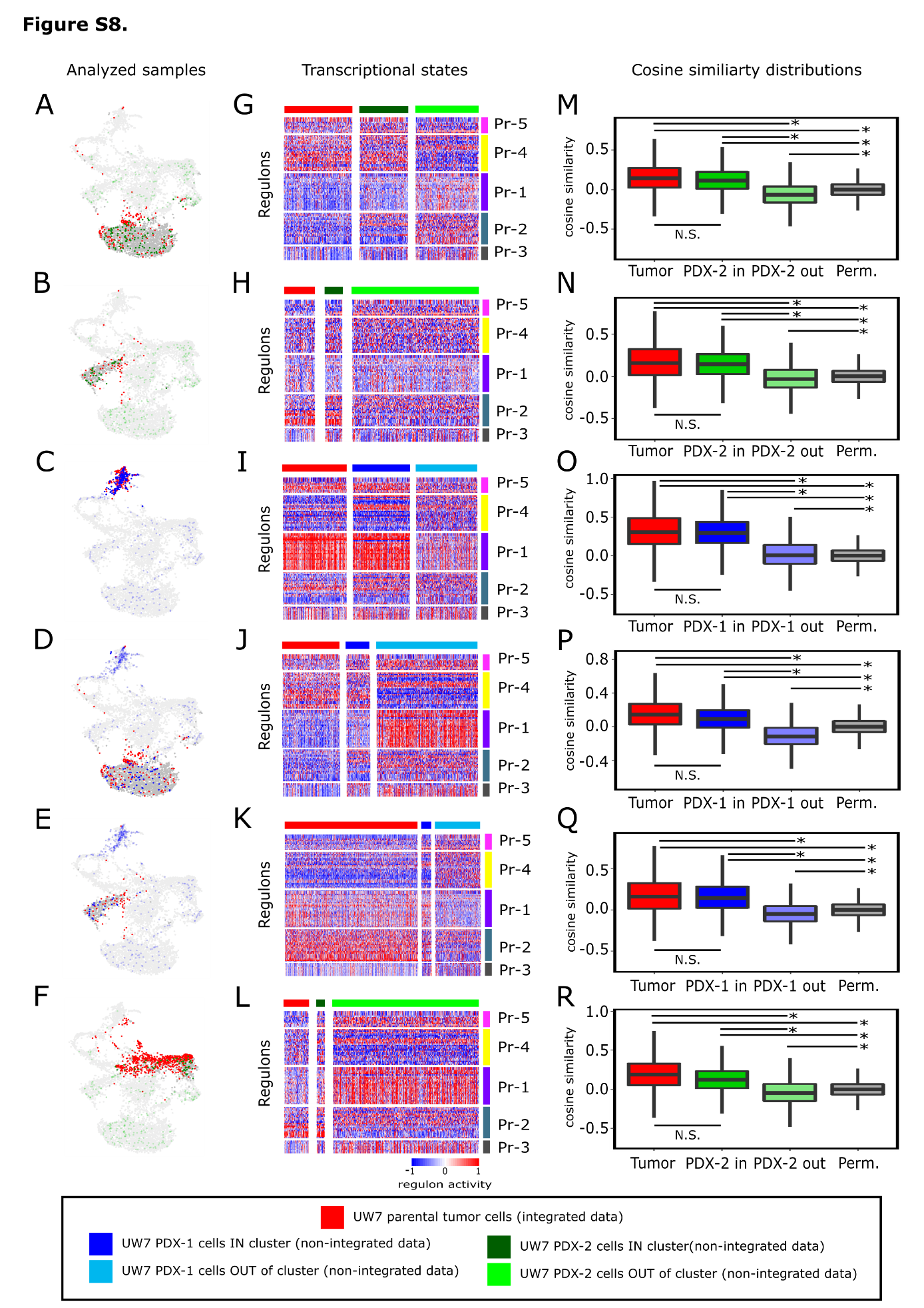


**Supplementary Figure 10. Comparison of transcriptional states in (batch-integrated) UW7 primary tumor snRNA-seq profiles and (non-integrated) PDX snRNA-seq profiles. (A-F)** UMAP plots of integrated primary and PDX tumor cells. Dark grey dots represent SNN-cluster being analyzed. Colored dots represent primary and untreated PDX tumor cells being compared. **(G-L)** Heatmaps of regulon activity (rows) that comprise transcriptional programs (Fig. 1E), the amalgamation of which define transcriptional network states of single cells (columns). The left portion of a heatmap represents network states of UW7 primary tumor cells determined via network inference analysis of batch-integrated (UW7 primary and PDX tumor cell) snRNA-seq dataset within a particular SNN-cluster. Using those same regulon genes, regulon activities were determined from the non-integrated PDX snRNA-seq data (Methods) for PDX cells within the same SNN-cluster (middle portion). Regulon activities for untreated PDX cells outside of the SNN-cluster of interest are included in the right portion of the heatmap. **(M-R)** Cosine similarity values determined from pairwise comparisons of transcriptional network states of primary tumor cells to (Tumor) one another within an SNN-cluster of interest, (PDX-# in) network states of untreated PDX tumor cells (non-integrated PDX snRNA-seq data) within the same SNN-cluster, and (PDX-# out) network states of untreated PDX cells (non-integrated PDX snRNA-seq data) outside of the SNN-cluster. The final boxplot (Perm.) represents the null distribution of cosine similarities based on randomly permuted versions of the batch-integrated data sets and non-integrated PDX tumor data. The centerline and bounds of the box for each boxplot represent the 50^th^, and 25^th^/75^th^ percentile of the cosine similarity values, respectively. Whiskers capture ±1.5 * interquartile range. Statistically significant distributions of cosine similarities (denoted by asterisks) indicate samples having distinct transcriptional states.

**Supplementary Figure 11.**


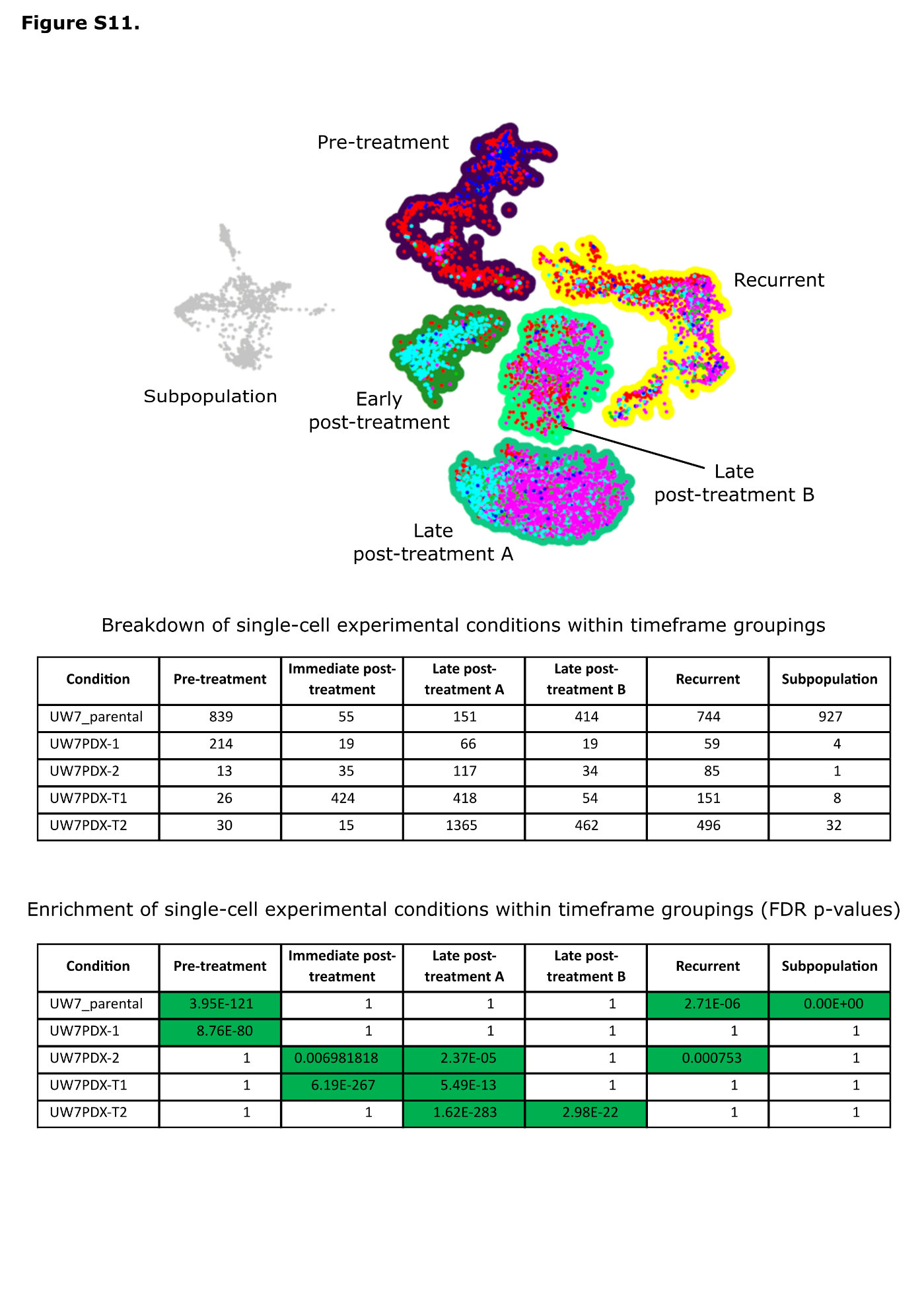


**Supplementary Figure 11. Enrichment of experimental conditions within longitudinal stages.** UMAP plot of single tumor cells and corresponding clusters identified based on PDX timepoints. Top table tabulates composition of each longitudinal stage with respect to experimental conditions within each stage. Bottom table tabulates corresponding FDR-adjusted *p*-values for enrichment of experimental conditions within each stage, per hypergeometric test. The “subpopulation” of tumor cells is a smaller subpopulation predominantly comprised of UW7 primary tumor cells (Supplemental text)

**Supplementary Figure 12.**


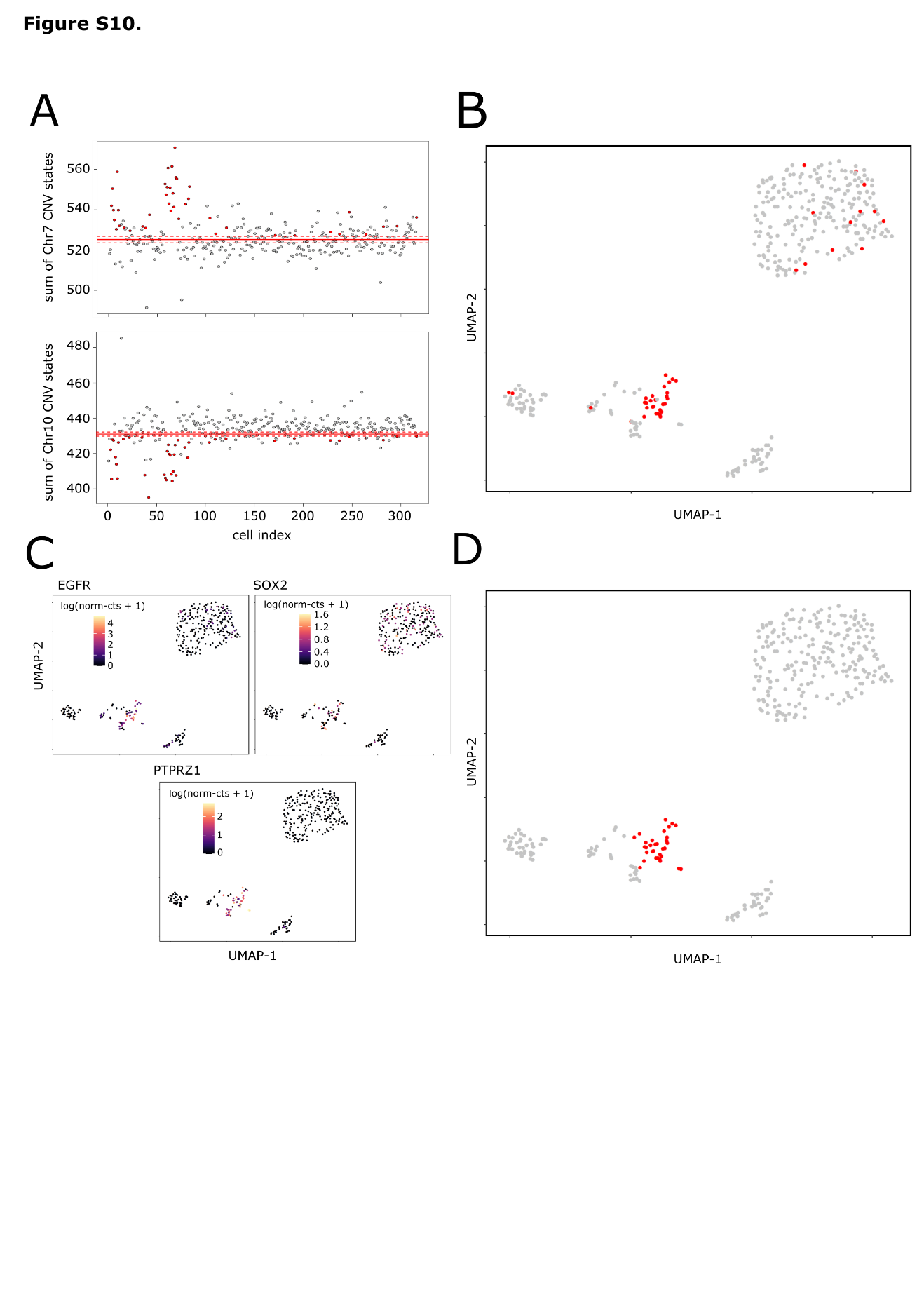


**Supplementary Figure 12. Tumor cell annotation in primary tumor biopsy.** **(A)** Sum of inferred copy number variation (CNV) states for Chr7 and Chr10 across 4,183 cells collected from primary tumor biopsy. Red solid lines represent the mean sum of inferred CNV state while dashed lines represent a 95% confidence interval of the average sum of inferred CNV state for reference neurons, classified as such per expression of neuronal markers RBFOX3 and MAP2. Red points represent cells having a Chr7 and Chr10 CNV state above and below, respectively, the confidence-level threshold based on CNV states of the reference cells. **(B)** UMAP plot of UW7 recurrent tumor snRNA-seq profiles. Red colored cells represent cells having an inferred CNV gain in Chr7 and loss in Chr10, shown in (A). **(C)** UMAP plot of UW7 recurrent tumor biopsy snRNA-seq profiles annotated according to log-normalized gene counts of tumor gene markers EGFR, SOX2, and PTPRZ1. **(D)** Final tumor-cell annotation of UW7 primary tumor cell. Based on inferred CNV state and the expression of tumor gene markers, 32 cells of the total 350 cells analyzed from the recurrent tumor biopsy were defined as tumor cells.

**Supplementary Figure 13.**


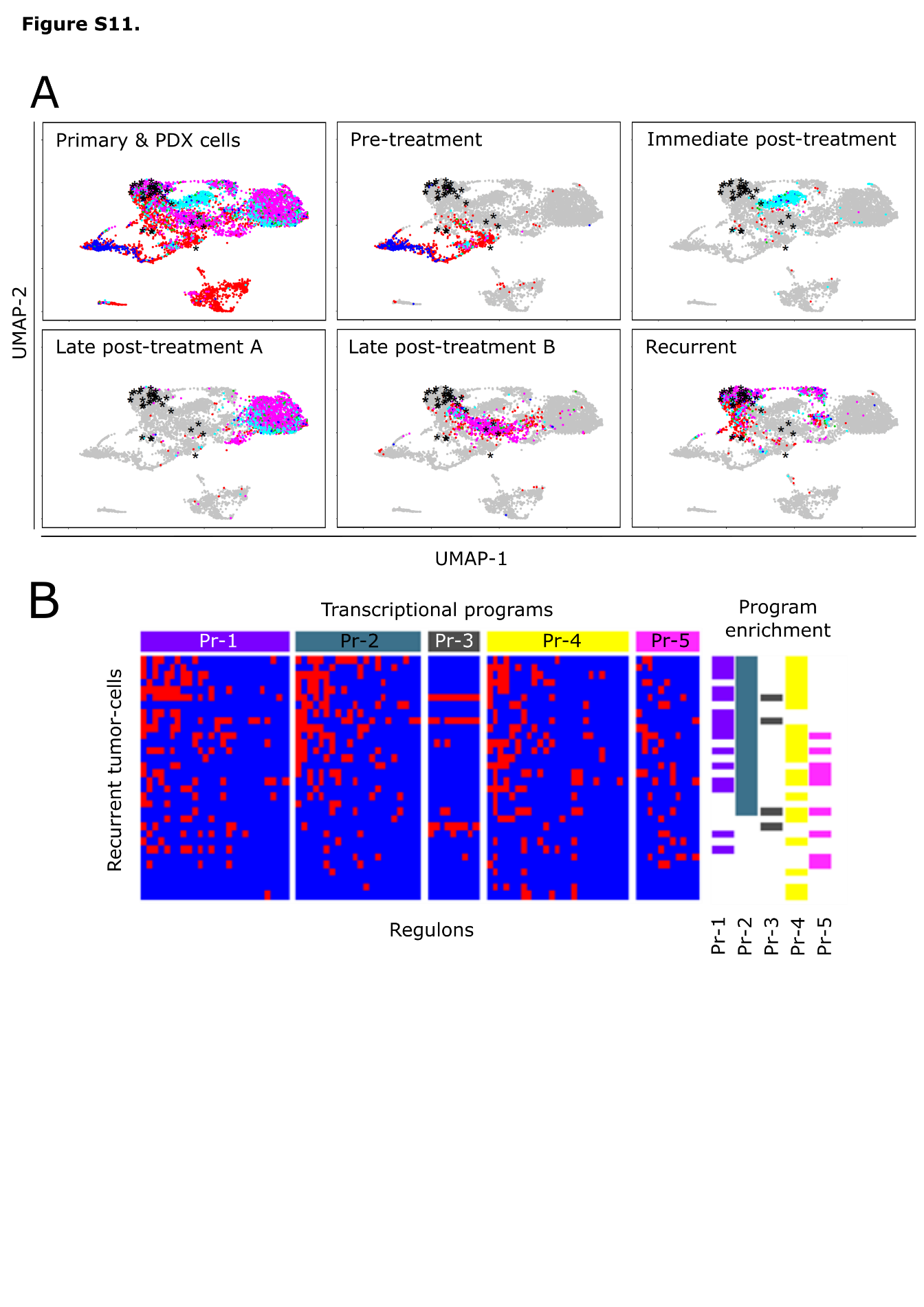


**Supplementary Figure 13. Projection of UW7 recurrent autopsy tumor-cell samples into lower dimensional space defined by primary and PDX tumor cells.** **(A)** UMAP plot of UW7 primary and PDX tumor cells based on common gene set shared across UW7 primary, PDX, and recurrent tumor cells collected at autopsy. Black asterisks represent projected UW7 recurrent tumor cells. Color annotation for UW7 primary/PDX tumor cells is identical to that of Fig. 3A. Subpanels highlight the primary and PDX tumor cells belonging to the various longitudinal stages (Fig. 3B). **(B)** Heatmap of *p*-values for associated enrichment scores of regulons in UW7 recurrent tumor cells. Here, *p*-values ≤ 0.1 are highlighted in red while *p*-values > 0.1 are in blue. Left-adjacent color bars mark recurrent tumor cells that exhibit statistical enrichment of regulons associated with a particular transcription program (Fig. 1E), as determined by a hypergeometric testing for enrichment of regulons having a positive and significant enrichment score.

**Supplementary Figure 14.**


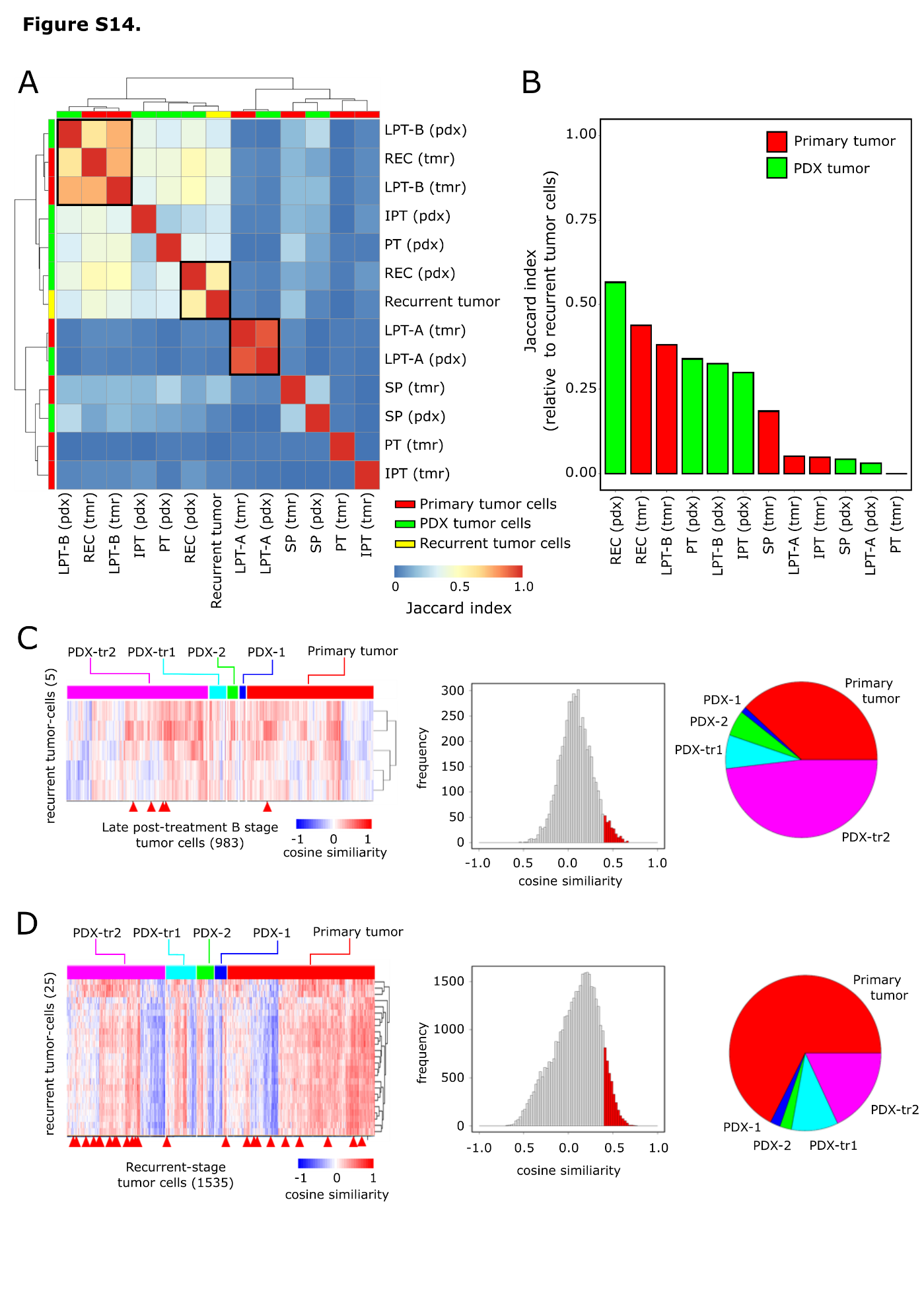


**Supplementary Figure 14. Phenotypic similarity of UW7 recurrent tumor cells to those in longitudinal stages.** **(A)** Heatmap of Jaccard indices quantifying pairwise similarity of phenotypic states among cell clusters. Black outlined pixels in heatmap indicate significant Jaccard indices (empirical *p*-value << 0.01, determined via permutation analysis involving 1000 random permutations of upregulated DEGs and subsequent gene set enrichment analysis). **(B)** Jaccard indices determined from pairwise of comparison of recurrent tumor cells to primary or PDX tumor cells within each longitudinal stage. **(C)** Heatmap of cosine similarity values of UW7 recurrent tumor samples that projected onto primary and PDX tumor cells within the LPT-B stage (Fig. 3B). Red arrowheads underneath the heatmap mark those UW7 primary/PDX tumor cells that were the closest neighbors to the projected UW7 recurrent tumor samples in the UMAP embedding space. Histogram visualizes the distribution of cosine similarity scores (based on Pr-2 activity per cell, which is highly active in a majority of tumor cells within the REC stage – Fig. 3B). The highlighted region indicate scores in the upper 90% quantile of the distribution. Adjacent pie graph indicates the proportion of UW7 primary/PDX tumor cells with which the recurrent tumor cells share cosine similarity within the upper 90% quantile of the cosine similarity distribution. **(D)** Similar set of graphs visualizing cosine similarity between UW7 recurrent tumor samples that projected onto UW7 primary/PDX samples within the REC stage (Fig. 3B).

**Supplementary Figure 15.**


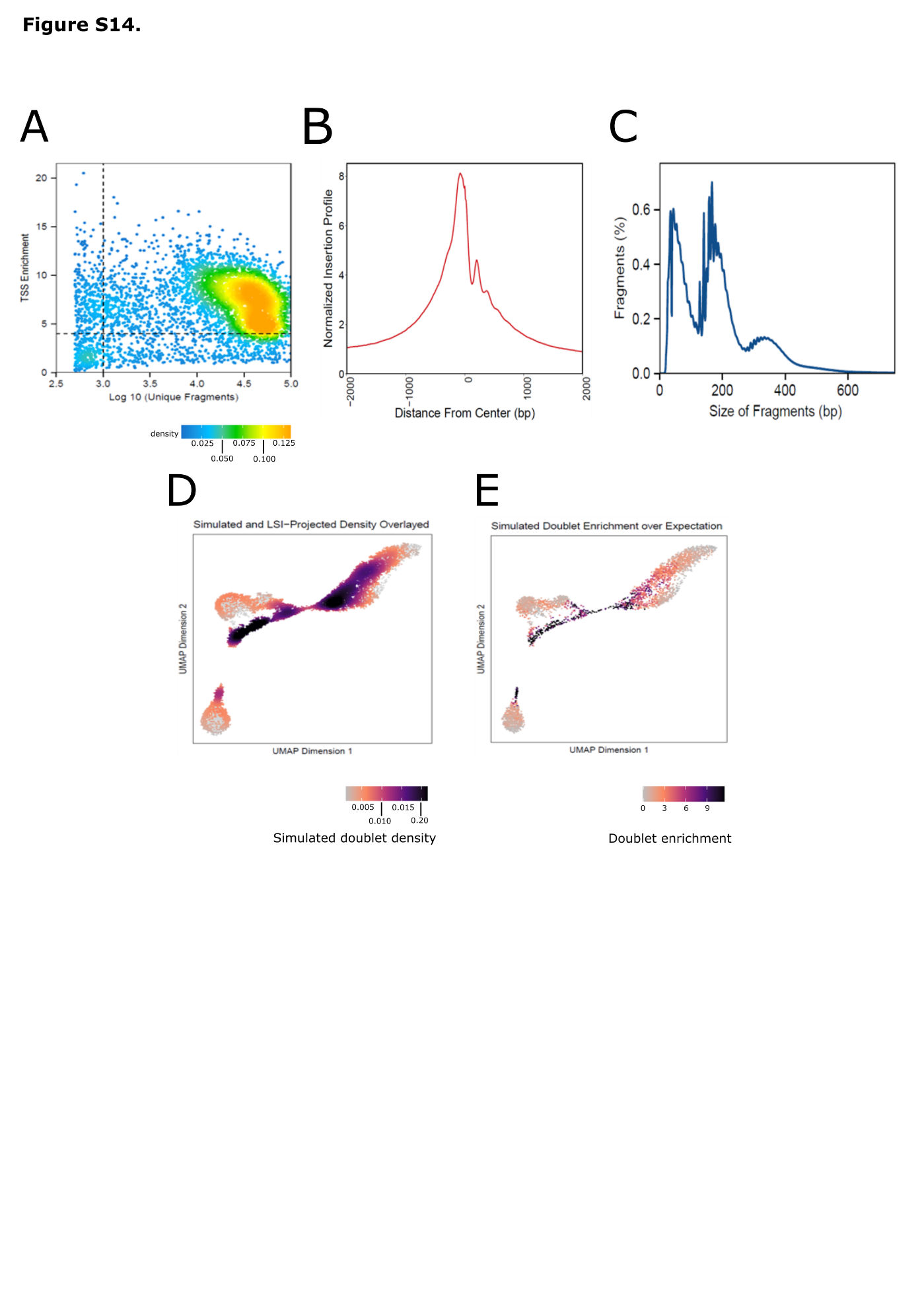


**Supplementary Figure 15. Quality control & doublet identification for snATAC-seq profiles from primary tumor biopsy.** **(A)** QC filtering plots for the UW7 primary tumor samples used for snATAC-seq analysis. Transcription start site (TSS) enrichment score vs. log-transformed unique nuclear fragments per cell. Here TSS enrichment score is a calculated value representing the signal-to-background ratio. Colors indicate density of points in the plot. Of the total cells analyzed, 3,531 cells passed QC filters, located in the upper right quadrant. **(B)** TSS insertion profiles centered at all TSS regions. **(C)** Fragment size distributions for the UW7 primary tumor cells passing ArchR QC thresholds. UMAP plots of snATAC-seq data visualizing **(D)** simulated doublet density and **(E)** simulated doublet enrichment.

**Supplementary Figure 16.**


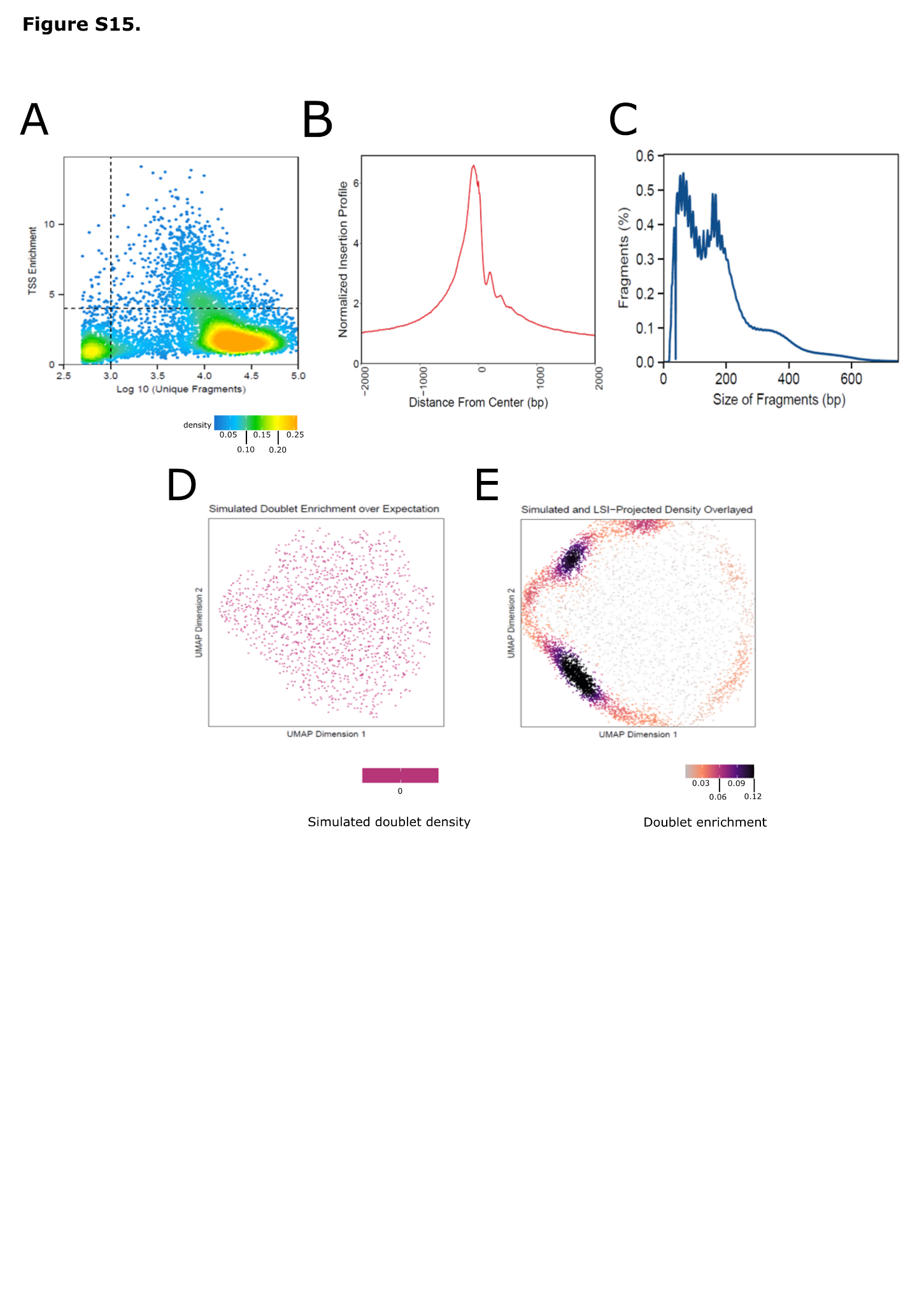


**Supplementary Figure 16. Quality control & doublet identification for snATAC-seq profiles from recurrent tumor biopsy.** **(A)** QC filtering plots for the UW7 recurrent tumor used for snATAC-seq analysis. Transcription start site (TSS) enrichment score vs. log-transformed unique nuclear fragments per cell. Colors indicate density of points in the plot. Of the total cells analyzed, 1425 cells passed QC filters, located in the upper right quadrant. **(B)** TSS insertion profiles centered at all TSS regions. **(C)** Fragment size distributions for the UW7 primary tumor cells passing ArchR QC thresholds. UMAP plots of snATAC-seq data visualizing **(D)** simulated doublet density and **(E)** simulated doublet enrichment.

**Supplementary Figure 17.**


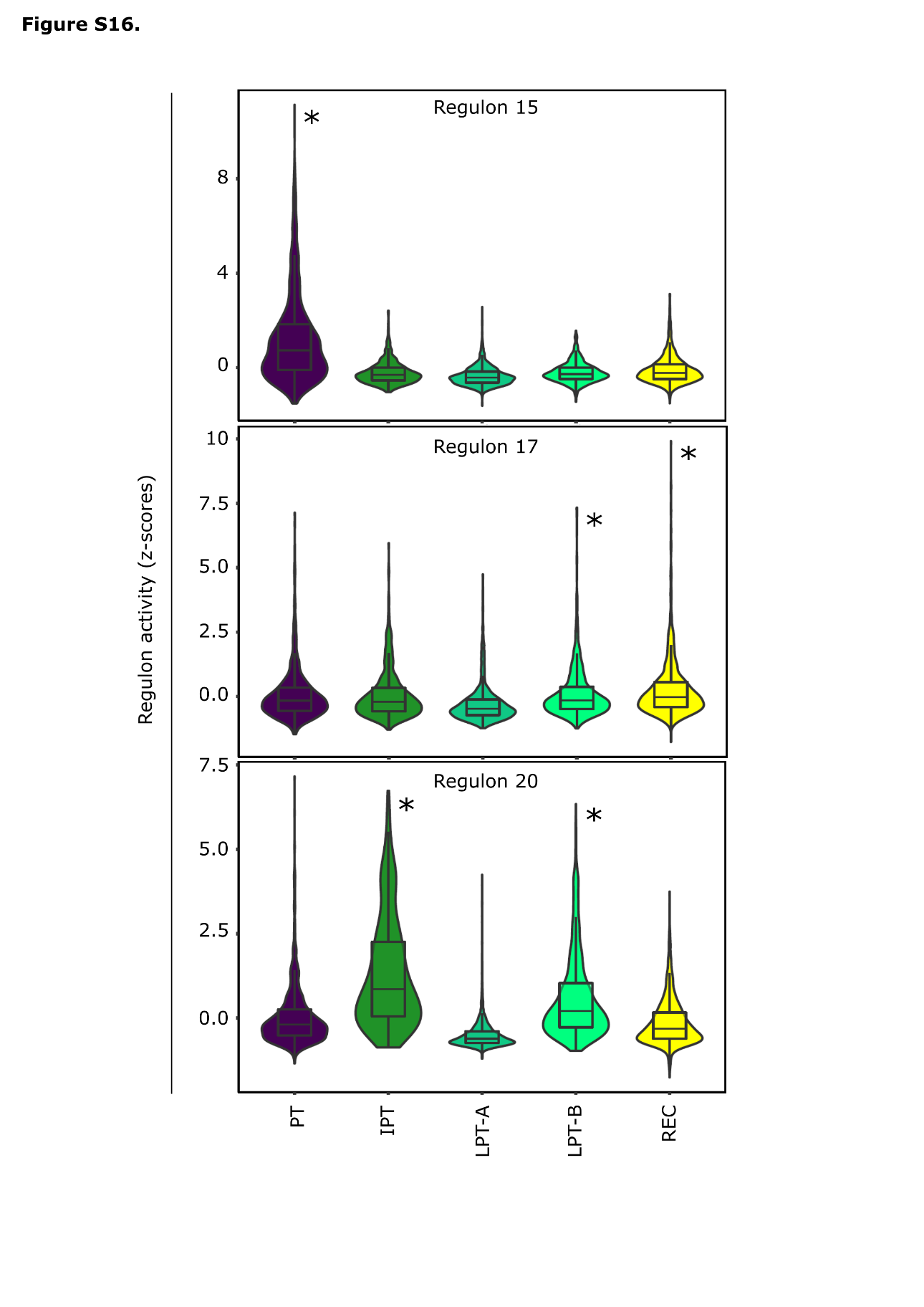


**Supplementary Figure 17. AR-regulated regulon activity across partitioned clusters.** Violin plots of regulon activity within each subpopulation for AR-regulated regulons 15, 17, and 20. Regulon 15 exhibited “selected against” behavior across the time course of treatment. Regulon 17 exhibited ‘selected/induced’ behavior and regulon 20 exhibited “transient” behavior. The centerline and bounds of the box for each boxplot represent the 50^th^, and 25^th^/75^th^ percentile of the cosine similarity values, respectively. Whiskers capture ±1.5 * interquartile range. Asterisks indicate which subpopulations had significantly higher regulon activity relative to the rest of the primary/PDX tumor-cell population per Wilcoxon rank sum test (FDR-adjusted *p*-value << 0.01).
