# Supplementary material for: A single-cell based precision medicine approach using glioblastoma patient-specific models
Source: NPJ Precis Oncol. 2022 Aug 8;6:55. doi: 10.1038/s41698-022-00294-4 (PMC9360428; doi:10.1038/s41698-022-00294-4)
Supplement: Supplementary file 2 — REPORTING SUMMARY [file 41698_2022_294_MOESM2_ESM.pdf]

nature portfolio

Corresponding author(s): Nitin S. Baliga, Anoop P. Patel

Last updated by author(s): Nov 26, 2021

Reporting Summary

Nature Portfolio wishes to improve the reproducibility of the work that we publish. This form provides structure for consistency and transparency in reporting. For further information on Nature Portfolio policies, see our Editorial Policies and the Editorial Policy Checklist.

Statistics

For all statistical analyses, confirm that the following items are present in the figure legend, table legend, main text, or Methods section.

n/a	Confirmed	
☐	☒	The exact sample size (n) for each experimental group/condition, given as a discrete number and unit of measurement
☐	☒	A statement on whether measurements were taken from distinct samples or whether the same sample was measured repeatedly
☐	☒	The statistical test(s) used AND whether they are one- or two-sided<br>*Only common tests should be described solely by name; describe more complex techniques in the Methods section.*
☐	☒	A description of all covariates tested
☐	☒	A description of any assumptions or corrections, such as tests of normality and adjustment for multiple comparisons
☐	☒	A full description of the statistical parameters including central tendency (e.g. means) or other basic estimates (e.g. regression coefficient) AND variation (e.g. standard deviation) or associated estimates of uncertainty (e.g. confidence intervals)
☐	☒	For null hypothesis testing, the test statistic (e.g. F, t, r) with confidence intervals, effect sizes, degrees of freedom and P value noted<br>*Give P values as exact values whenever suitable.*
☒	☐	For Bayesian analysis, information on the choice of priors and Markov chain Monte Carlo settings
☒	☐	For hierarchical and complex designs, identification of the appropriate level for tests and full reporting of outcomes
☒	☐	Estimates of effect sizes (e.g. Cohen's d, Pearson's r), indicating how they were calculated

Our web collection on statistics for biologists contains articles on many of the points above.

Software and code

Policy information about availability of computer code

Data collection	single-cell RNA-seq and single-cell ATAC-seq counts were obtained using the Cell Ranger Sinlge-Cell Software Suite, provided by 10X Genomics.
Data analysis	Regulatory network inference was performed using software developed and maintained by the Baliga Lab (MINER). Software can be found in the following github page - https://github.com/baliga-lab/miner3

For manuscripts utilizing custom algorithms or software that are central to the research but not yet described in published literature, software must be made available to editors and reviewers. We strongly encourage code deposition in a community repository (e.g. GitHub). See the Nature Portfolio guidelines for submitting code & software for further information.

Data

Policy information about availability of data

All manuscripts must include a data availability statement. This statement should provide the following information, where applicable:

- Accession codes, unique identifiers, or web links for publicly available datasets
- A description of any restrictions on data availability
- For clinical datasets or third party data, please ensure that the statement adheres to our policy

Data generated for this study is available through the Gene Expression Omnibus (GEO) under the accession codes GSE189650 (UW7 snRNA-seq) and GSE157910 (UW7 scATAC-seq).

Field-specific reporting

Please select the one below that is the best fit for your research. If you are not sure, read the appropriate sections before making your selection.

☒ Life sciences ☐ Behavioural & social sciences ☐ Ecological, evolutionary & environmental sciences

For a reference copy of the document with all sections, see nature.com/documents/nr-reporting-summary-flat.pdf

Life sciences study design

All studies must disclose on these points even when the disclosure is negative.

Sample size	This study was designed to investigate disease progression of a single glioblastoma patient via single-cell analysis of an individual patient tumor, corresponding patient-derived xenografts from the same patient, and corresponding recurrent tumor from same patient. The number of single-cell samples analyzed was dependent on the experimental constraints and quality control filtering of samples, as described in the Methods section of the manuscript.
Data exclusions	Single-cell omic profiles that were excluded were those samples that did not meet pre-defined quality control standards, as described in the Methods section of the manuscript.
Replication	This study was a characterization and analysis of an individual patient's tumor throughout its progression and post-treatment evolution (as modeled by patient-derived xenografts). Because this study focused on an individual patient, we were unable to replicate the results due to the inherent uniqueness of a the patient's tumor. In addition, lack of sample availability for another patient's tumor throughout its disease progression prevented replication of the analytical approach used in this study.
Randomization	Because this study focused on developing a framework to model and analyze tumor progression of a single patient, there was no need for randomization of this study.
Blinding	Because this study focused on developing a framework to model and analyze tumor progression of a single patient, there was no need for blinding in this study.

Reporting for specific materials, systems and methods

We require information from authors about some types of materials, experimental systems and methods used in many studies. Here, indicate whether each material, system or method listed is relevant to your study. If you are not sure if a list item applies to your research, read the appropriate section before selecting a response.

Materials & experimental systems

n/a	Involved in the study	
☒	☐	Antibodies
☒	☐	Eukaryotic cell lines
☒	☐	Palaeontology and archaeology
☒	☐	Animals and other organisms
☐	☒	Human research participants
☒	☐	Clinical data
☒	☐	Dual use research of concern

Methods

n/a	Involved in the study	
☒	☐	ChIP-seq
☒	☐	Flow cytometry
☒	☐	MRI-based neuroimaging

Human research participants

Policy information about studies involving human research participants

Population characteristics	This work involved an N-of-1 analysis of a patient tumor throughout disease progression and treatment-induced evolution. No population characteristics were included.
Recruitment	Tumor biopsy samples from this N-of-1 patient study were collected as part of an intraoperative tumor specimens from adult patients who voluntarily consented to donation to the institutional tumor bank.
Ethics oversight	Institutional Review Board (IRB)-approved protocols (protocol #STUDY00002162)

Note that full information on the approval of the study protocol must also be provided in the manuscript.
